# Microfluidic lung cancer models: Bridging clinical treatment strategies and tumor microenvironment recapitulation

**DOI:** 10.1063/5.0282002

**Published:** 2025-12-16

**Authors:** Zhiyun Yu, Arsalan A. Khan, Wara Naeem, Jeffrey A. Borgia, Michael J. Liptay, Christopher W. Seder, Jian Zhou

**Affiliations:** 1 Department of Cardiovascular and Thoracic Surgery, Rush University Medical Center, 1725 W Harrison Street, Chicago, Illinois 60612, USA; 2 Department of Anatomy and Cell Biology, Rush University Medical Center, 1735 W Harrison St, Chicago, Illinois 60612, USA; 3 Department of Pathology, Rush University Medical Center, 1750 W Harrison St, Chicago, Illinois 60612, USA

## Abstract

Lung cancer remains the leading cause of cancer-related mortality worldwide, with non-small cell lung cancer accounting for a majority of cases. Despite advances in targeted therapies and immunotherapy, challenges such as tumor heterogeneity, resistance mechanisms, and limited preclinical models hinder treatment efficacy. Traditional cancer models, including 2D cell cultures and animal models, often fail to accurately replicate the lung's complex architecture, microenvironment, and biomechanical cues, leading to poor predictive performance in drug development. Microfluidic-based organ-on-a-chip technology offers a promising alternative by integrating human-derived cells with precisely controlled perfusion, mechanical cues, and tumor–stroma interactions in physiologically relevant 3D models. These platforms enable the study of lung cancer biology, drug responses, and patient-specific therapeutic outcomes with improved accuracy. In this review, we discuss recent advancements in microfluidic systems for recapitulating normal lung physiology and 3D lung cancer microenvironment, covering various microfluidic platforms with applications in disease modeling and drug testing. Unlike other review articles, we bring first-hand insights from clinicians about the current treatment practice for lung cancer and the clinical utilities of lung cancer-on-a-chip models, which bioengineers have been seeking. We also highlight the translational potential of these systems in personalized oncology and the need for interdisciplinary collaborations, particularly with clinicians, to enhance their clinical impact.

## I. INTRODUCTION

Lung cancer is the leading cause of cancer-related deaths worldwide, with approximately 1.8 × 10^6^ deaths annually.^1,2^ About 85% of cases are non-small cell lung cancer (NSCLC), primarily lung adenocarcinoma (LUAD), and lung squamous cell carcinoma (LUSC).^3^ About two-thirds of lung cancer deaths worldwide are attributable to smoking, particularly in regions where smoking is prevalent, with stronger associations in LUSC and small cell lung cancer (SCLC) than LUAD. However, lung cancer in never smokers is linked to environmental factors like secondhand smoke, pollution, occupational carcinogens, and genetic predisposition.^1^ The development of targeted therapies and the successful application of immunotherapy in select patient populations have marked significant progress in treating advanced NSCLC. However, challenges remain, including the need to identify new driver gene alterations to broaden the scope of targeted therapies and to better understand resistance mechanisms to improve their long-term effectiveness.

Lung cancer exhibits significant intratumoral heterogeneity due to the presence of sub-populations of cells with distinct molecular features. An increased sub-clonal mutation fractions in localized LUAD are linked to a higher risk of post-surgical relapse, suggesting early metastatic potential in heterogeneous tumors.^3^ Thus, a personalized treatment regimen is necessary to identify the clonal targetable genetic alterations early in diagnosis and to timely monitor the emergence of post-therapy acquired resistant targets.

Traditional models like animal systems and cancer cell lines are widely used to study anticancer drugs, offering cell-type-specific mechanistic insights. Animal cancer models, including patient-derived xenograft models, genetically engineered mouse models, and microsurgical tumor cell injection models, require extended establishment time ranging from three months to over a year.^4^ Despite their utility, these models often fail to fully capture the intratumoral heterogeneity observed in individual patients. While simple 2D *in vitro* models focus on molecular mechanisms, but they failed to replicate the human lung's complex architecture and physiological functions, including the alveolar–capillary barrier, breathing motion-induced mechanistic force, extracellular matrix (ECM), and multiorgan interactions, resulting in poor predictive performance and high drug development failure rates.^5^ Developing advanced, accurate, and efficient models is essential for improving anticancer drug evaluation and success rates (Box 1).

BOX 1 BOX 1. Clinical significance of lung cancer models.

From a purely clinical standpoint, the ability to simulate the true physiological environment of the lung would represent a significant advancement in managing lung cancer. In addition to evaluating the response to adjuvant therapy—delivered post-surgical resection—using cells from biopsy samples, we can also monitor the evolution of the tumor's genomic profile and its immune microenvironment. This would enable the identification of targetable mutations and the tailoring of treatments to achieve the most optimal and effective therapeutic outcomes.

Furthermore, understanding how tumor cells influence the immune microenvironment and interact with the blood vessel endothelium would help assess the effectiveness and bioavailability of immunotherapy drugs, such as PD-L1 inhibitors. Clinicians face the challenging responsibility of balancing the benefits and harms of chemotherapy and immunotherapy to ensure patient safety. The most serious side effects include organ toxicities, such as hepatotoxicity, pneumonitis, cardiac toxicity, and neurological dysfunction. With this technology, the effects of chemotherapeutic drugs on other cells and organs within the body could be studied in a patient-specific manner, allowing for the prediction of potential toxicities before treatment begins.

Additionally, combination therapies involving multiple drugs could be assessed rapidly on patient-derived samples, reducing trial-and-error approaches on the patient themselves and thereby enhancing patient safety. Such insights would help in preventing the development of drug resistance and would enable a more personalized and safer approach to lung cancer management.

Organ-on-a-chip, or microphysiological systems, integrate human-derived cellular material with microfluidic technology to recreate the *in vivo* microenvironment of organs *in vitro.*^6^ These platforms mimic key physiological parameters, such as perfusion and mechanical forces, offering a controlled and biologically relevant system for drug testing and disease modeling. Microfluidic chips provide precise control of fluid flow within small channels seeded with cells in 2D and 3D formats. For lung cancer research, these chips enable the integration of 3D co-cultures, nutrient and gas supply, shear stress, tensile forces, drug concentration gradients, and real-time biosensing to monitor cellular behaviors under various conditions. 3D organoid models in microfluidic systems have been used to elucidate cancer biology and facilitate drug testing.^7^ Preclinical organoid models integrated with these systems are increasingly being explored for predicting patient responses to treatment.^8^

**FIG. 1.**
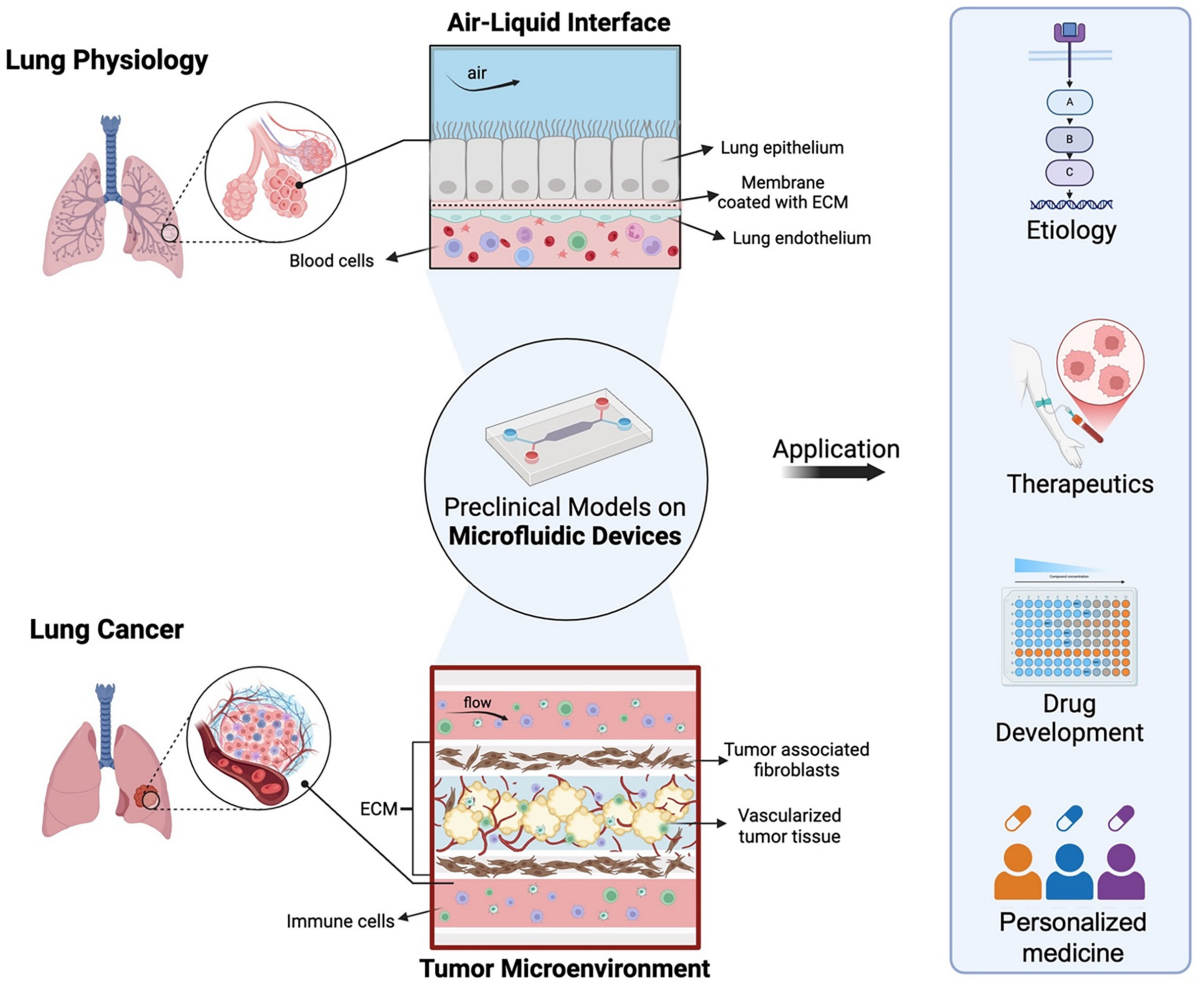
Microfluidic lung cancer models bridging lung physiology and cancer biology for precision lung cancer care. Modeling lung architecture and physiology in microfluidic devices enables readily recreation of key features of the lung tumor microenvironment, including complex cellular compositions and vascular networks. These lung and lung cancer-on-a-chip systems have broad applications in disease etiology, therapeutics, drug development, and personalized medicine.

This review highlights advancements in microfluidic systems for modeling normal lung tissue and lung cancer (Fig. 1). It begins by outlining the current challenges in lung cancer management within clinical practice. The subsequent sections delve into microfluidic fabrication techniques and chip design for lung cancer research, followed by a discussion of recent models of lung-on-a-chip and lung cancer-on-a-chip, which excel at replicating tissue microenvironments and hold promise for clinical diagnostics and drug development. Finally, the review addresses the critical role of cross-disciplinary collaboration among scientists, bioengineers, and clinicians, along with the challenges inherent in fostering such partnerships.

## II. CURRENT LANDSCAPE IN CLINICAL MANAGEMENT OF LUNG CANCER

Due to the emergence of advanced therapeutic modalities, the treatment landscape for NSCLC has advanced significantly; however, improving survival outcomes remains a challenge. About half of NSCLC cases are diagnosed at stages I–III, and even in these cases, the 5-year disease-free survival (DFS, refer to Table I for common terms) ranges from 34% to 80%, improving from stage III to stage I disease.^9–11^ Additionally, 5-year overall survival (OS) for resectable NSCLC ranges from 50% to 70%.^12,13^

**TABLE I.** Nomenclature in cancer research and care.

Terminology	Definition
5-year overall survival	Refers to the percentage of patients who are still alive after 5 years of follow-up
5-year recurrence-free survival rate	Represents the percentage of patients who have not experienced a recurrence of NSCLC within 5 years
Disease-free survival	Refers to the length of time a cancer patient survives without any signs or symptoms of cancer after treatment
Comorbidities	Comorbidities are medical conditions or diseases that coexist with a primary condition in a patient
Adjuvant therapy	Additional therapy provided after primary treatment to reduce the risk of disease recurrence
Neo-adjuvant therapy	Additional therapy administered before primary treatment, such as surgical resection, to reduce tumor size and spread, facilitating more effective removal
Tumor downstaging	Reduction in tumor stage achieved through neoadjuvant therapy
Immune checkpoint proteins	Proteins that act as checkpoints in the immune system and downregulate immune system responses to stimuli; most cancers exploit these proteins to evade immune detection
Immune checkpoint inhibitors	Monoclonal antibodies that target checkpoint proteins to restore immune responses against cancer; widely used as immunotherapy in NSCLC
Pathologic complete response (pCR)	Refers to the presence of zero viable cancer cells in the resected tumor and regional lymph nodes after neoadjuvant treatment; assessed via pathological examination of tissue post-treatment
TNM stage	Stands for tumor, node, and metastasis. It is a system that describes the size and spread of a tumor in a patient's body.

### A. Overview of disease management plan

Early-stage NSCLC (stages I–IIIA) is generally considered resectable, except in cases where stage IIIA patients have mediastinal lymph node involvement or bulky/matted lymph nodes.^11^ Conversely, unresectable cases are usually advanced cancers, the majority of which are bulky stage IIIB–IV tumors with metastases.^14,15^ In addition to advanced stage, several factors can make a tumor unresectable. These include proximity to major blood vessels like the superior vena cava, and location near the carina, where the bronchi divide. Furthermore, poor lung function from conditions like chronic obstructive pulmonary disease, severe cardiac disease, advanced age with significant comorbidities, or inability to tolerate major surgery can make surgical intervention inadvisable. This underscores the need for ongoing innovation in the multidisciplinary management of resectable NSCLC.

### B. Treatment of early-stage NSCLC

#### 1. Surgical resection and adjuvant therapy

**FIG. 2.**
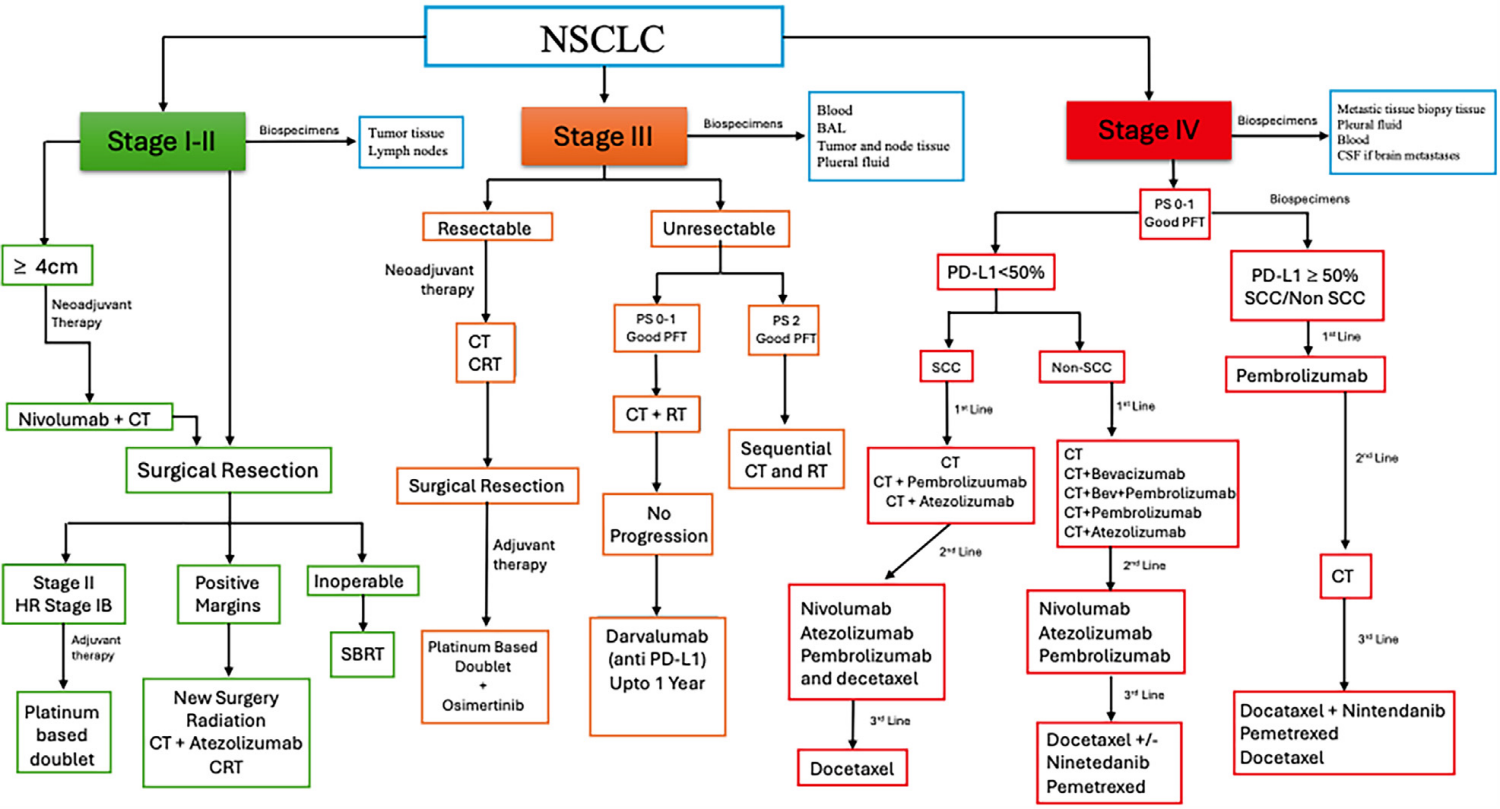
Current treatment guidelines for non-small cell lung cancer (NSCLC). BAL: bronchoalveolar lavage; CT: chemotherapy; HR: high risk; CRT: chemoradiotherapy; SBRT: stereotactic body radiation therapy; RT: radiotherapy; PD-L1: programmed death-ligand 1; SSC: squamous cell carcinoma; Bev: bevacizumab pulmonary function test; PS: performance status, which is a standardized score, ranging from 0 (fully active) to 5 (deceased: PFT) used to assess fitness for surgery, with scores ≤2 generally indicating suitability for surgery.

The current treatment guidelines for NSCLC are determined based on cancer's stage, genetic mutations, and the patient's overall health (Fig. 2). Historically, surgical resection was the primary treatment for early-stage NSCLC, though recurrence rates were high, approaching 50%.^16^ The advent of cisplatin-based adjuvant chemotherapy marked a critical development by significantly reducing recurrence and improving survival.^17^ The Lung Adjuvant Cisplatin Evaluation (LACE) meta-analysis found modest yet notable improvements in 5-year DFS and overall survival (OS), increasing by 5.8% and 5.4%, respectively.^18^ However, adjuvant chemotherapy has seen limited uptake due to poor compliance, as many patients struggle to complete treatment after surgery.^19^

#### 2. Neoadjuvant therapy

Neoadjuvant chemotherapy has recently gained favor due to its potential for higher compliance, tumor downstaging, and enabling more complete resections.^20^ It also provides valuable opportunities for translational research, such as biomarker identification and tumor response assessment.^20^ Integrating immune checkpoint inhibitors (ICIs) with neoadjuvant therapy has transformed the field, with trials like CheckMate 816, KEYNOTE-671, and CheckMate 77T,^21–23^ which showed improved pathologic complete response (pCR) and event-free survival (EFS) when combining chemotherapy with immunotherapy. These findings support chemoimmunotherapy as the preferred induction regimen for patients without actionable gene alterations.^24^ Immunotherapy benefits are also evident in adjuvant settings, raising the question of whether adjuvant or neoadjuvant administration offers better outcomes.^25^ Table II shows the systemic therapy regimens for the perioperative management.

**TABLE II.** Systemic therapy regimens currently in use for the perioperative management of non-small cell lung cancer.

Regimen	Action of mechanism
**Neoadjuvant or adjuvant chemotherapy**	
Cisplatin + pemetrexed (non-squamous)	Induces DNA damage; inhibits nucleotide synthesis via targeting folate metabolism
Cisplatin + gemcitabine (squamous)	Induces DNA damage; inhibits DNA synthesis and repair
Cisplatin + docetaxel (squamous)	Induces DNA damage; induces microtubules depolymerization; induces apoptosis; interferes angiogenesis and tumor microenvironment interactions
**Adjuvant targeted therapy**	
Alectinib (ALK rearrangements)	Inhibits ALK fusion proteins; inhibits PI3K-AKT, MAPK-ERK, JAK-STAT pathway
Osimertinib (EGFR mutations)	Inhibit EGFR with mutations including Del19, L858R mutation, and T790M; inhibits PI3K-AKT, MAPK-ERK, JAK-STAT pathway
Atezolizumab (IV) PD-L1	Monoclonal antibodies to block PD-L1 ligand on tumor cell surface; enhances T cell-mediated immune killing of cancer cells
Durvalumab	
Pembrolizumab	Monoclonal antibodies to block PD-1 receptor on T-cell surface; enhances T cell-mediated immune killing of cancer cells
Nivolumab	
**Neoadjuvant chemo-immuno therapy**	
Nivolumab + platinum doublet chemotherapy	Chemotherapy reduces tumor burden and releases tumor antigens; immunotherapy enhances T cell-mediated killing of cancer cells
Pembrolizumab + cisplatin-based doublet chemotherapy	
Durvalumab + platinum doublet chemotherapy	

#### 3. Targeted and immuno-therapies

For patients with actionable mutations in genes such as epidermal growth factor receptor (EGFR) or anaplastic lymphoma kinase (ALK), targeted therapy has become a cornerstone of NSCLC management.^26^ However, the optimal timing—preoperative vs postoperative—remains under study.^23^ While preoperative administration may elicit a stronger immune response, further trials are needed to confirm long-term benefits. Targeted therapies, such as osimertinib for EGFR-mutant NSCLC and alectinib for ALK-rearranged tumors, have shown significant DFS gains in the adjuvant setting.^23,26,27^ Trials like NeoADAURA and ALNEO are now evaluating these agents in neoadjuvant phases, potentially reshaping treatment protocols.^28,29^

### C. Management of advanced and metastatic NSCLC

According to the National Comprehensive Cancer Network (NCCN) guidelines, management of advanced and metastatic NSCLC is complex. The mainstay of treatment involves tumor-specific targeted therapy, often with concurrent chemoradiation, depending on the tumor's TNM stage and the extent of metastases. Molecular testing is essential to identify potentially targetable mutations in genes such as EGFR, ALK, KRAS, ROS1, BRAF, NTRK1/2/3, MET (exon 14 skipping), RET, and ERBB2 (HER2), and for assessing PD-L1 expression levels, which guide further treatment decisions. For EGFR, this includes detecting specific mutations such as exon 19 deletions, exon 21 L858R mutations, S768I, L861Q, G719X mutations, and exon 20 insertions.

### D. Unmet clinical need

Currently, approved targeted therapies include drugs like osimertinib, afatinib, erlotinib, larotrectinib, and selpercatinib (LOXO-292), among others.^30^ Recommended treatment regimens often combine chemotherapy, radiation, immunotherapy, and sometimes surgery. However, a common challenge clinicians face is that both targeted and non-targeted therapies, while initially effective, often succumb to acquired drug resistance due to factors like epigenetic mutations or tumor heterogeneity.^31–33^ This underscores the need to develop new targeted therapies, identify drug response, and necessary therapy adjustments *in vivo* for more effective management. Therefore, treating advanced-stage NSCLC is akin to addressing a moving target.^34^ Advancing personalized care in NSCLC requires a collaborative, interdisciplinary approach to effectively integrate emerging therapies into clinical practice. Such a collaboration is particularly manifested in establishing preclinical models for lung cancer disease management.

## III. MICROFLUIDIC PLATFORMS FOR LUNG CANCER RESEARCH

Microfluidic platforms for lung cancer studies hold great promise for advancing personalized medicine by closely mimicking the physiological and pathological conditions of lung cancer in preclinical models. These systems effectively replicate respiratory mechanical motion, tumor tissue architecture, and dynamic fluid flow, thereby recreating *in vivo* conditions. Furthermore, the integration of patient-derived cells/microtissues and the high-throughput capabilities of these chips enable time- and cost-effective evaluations of treatment efficacy tailored to individual patients.

### A. Microfabrication for tissue engineering

The microfluidic technology enables processing or manipulating small (10^−9^–10^−18^ l) amounts of fluids and biological tissues using channels and microwells with dimensions of tens to hundreds of micrometers.^35^ Conventional fabrication methods, such as laser micromachining, molding, and imprinting, have been foundational in microfluidic device production.^36^ However, the growing demands of biomedical research—requiring higher precision, flexibility, and scalability—have driven the adoption of advanced techniques like photolithography, soft lithography, and 3D printing.^36,37^ Photolithography employs ultraviolet (UV) light to transfer geometric patterns from a photomask to a light-sensitive photoresist on substrates like silicon or glass. Its high resolution, precision, and reproducibility make it ideal for high-volume manufacturing.^38^ Soft lithography, on the other hand, uses elastomeric materials, such as polydimethylsiloxane (PDMS), to create stamps, molds, or devices with fine features replicated from master molds, which are often made from photolithography, milling, or 3D printing. PDMS adhesive-based techniques further allow tuning of bonding strength to meet specific application needs.^39^ This approach supports the fabrication of three-dimensional, multiple layer, and complex microstructures. Its low cost, fast turnaround, and cleanroom-free process make it well-suited for rapid prototyping and small-scale production.^38^ While PDMS is biocompatible and optically transparent, the hydrophobicity of this material makes it difficult for direct cell culture, which requires appropriate surface coating for proper cell adherence.^40^ Recently, 3D printing has gained traction in biomedical tissue engineering. Techniques such as fused deposition modeling (FDM) and digital light processing (DLP) enable the layer-by-layer additive manufacturing of structures.^36^ 3D printing's flexibility allows for the creation of complex, customized designs without molds, facilitating the replication of intricate tissues containing multiple cell types, such as vascular grafts, skin, bone, and heart tissue.^41^ However, FDM-based 3D printing is limited by resolutions of hundreds of micrometers, while other methods are costly and offer limited material options.^36^ Despite these advancements, soft lithography remains the most widely used fabrication method for microfluidic devices in research settings due to its accessibility, versatility, and cost-effectiveness.

Microfluidic systems are well suited for biomedical research. A microfluidic system for cell biology research has several key components: (1) methods of introducing fluids, gases, and cell samples; (2) methods for controlled moving and mixing these fluids, gases around on the chip; (3) methods for realizing the microanalytical activities (e.g., imaging, biosensors) on the chip.^35^ Compared to conventional cell biology systems, these microsystems are uniquely advantageous for cell biology studies. In the case of fluid flow, the effect of the inertial forces to frictional forces can be reduced in microsystem, leading to the formation of laminar flow in the microfluidic channels. This property enables the formation of static and dynamic gradients at subcellular resolution, which are critical factors in regulating various biological processes, such as immune response, cancer spread, and cell differentiation.^42,43^ The rapid diffusive heat and mass transfer at the microscale, with characteristic times ranging from approximately 10^−3^ to 1 s (compared to 10^2^–10^4^ s at the macroscale), allows for quick media and environmental changes as well as efficient temperature control.^39^ The utility of a hypoxic incubator along with side gas channels, gas-permeable materials, and chemical deoxygenation on chips, recreate the intricate gaseous environment of lung and the tumor by enabling the flexible control of oxygen concentration and gradients.^44–46^ Other advantages include low consumption of reagents and cellular materials, simultaneous, independent, high-throughput manipulation of cells, and simulation of the *in vivo* cellular microenvironment. The design of a microfluidic chip must meet the need of rendering the cellular behavior and physiological environment, favoring the experimental practice of revealing pathological process and drug response.

### B. Engineering principles and strategies of microfluidic lung and lung cancer models

The primary principle of microfluidic design is to reproduce the complexity in cellular architecture and microenvironment. The varying geometry of lung trachea, alveoli, and tumor as well as their cellular composition need to be considered. Recreating the lung barrier, composed of epithelial and endothelial layers, typically requires channels or wells that separate air and media compartments while maintaining access to both. Intricate channel designs, combined with flow perfusion, simulate blood flow and shear stress within the lung's microvascular network. Cyclic stretching of alveoli during breathing can be mimicked by pressure-driven deformation of elastic materials integrated into the microsystem.^47^ The ability to precisely control gradients and spatiotemporal variations of gases and fluids positions microfluidic lung models as powerful tools for disease modeling, drug screening, and personalized medicine. The most typical microfluidic designs mimicking lung physiological components can be categorized into membrane-based platform, lumen platform, microchannel platform, and microwell platform. The design details of these various platforms have also been covered in recent reviews.^45,48^

The membrane-based platform is widely used to model airway epithelium and alveoli structures, featuring upper and lower channels separated by a porous membrane.^49^ The epithelial and vascular cells can be seeded on opposite sides of the membrane, and the pores on membrane allow the crosstalk and migration of different cell types. A defining characteristic is the air–liquid interface (ALI) created by air perfusion in the upper channel and medium supply in the lower channel, effectively mimicking airway tissue architecture. This ALI-based design underpins many lung-on-a-chip platforms, facilitating studies of infection and respiratory pathologies.^50,51^ A notable modification of ALI-based lung cancer-on-chip models involves implanting cancer cell lines into the airway or alveolar epithelial layer and simulating breathing movements using cyclic mechanical strain.^47^ This study revealed that mechanical breathing motion significantly influences NSCLC growth. Under static ALI conditions, tumor cell growth and invasion into the endothelial layer were enhanced. In contrast, simulated breathing motion markedly suppressed tumor cell migration.

The lumen platform features hydrogel-filled channels and is commonly used for vascularizing tumor cell–hydrogel mixtures or tumor spheroids/organoids. Lee *et al.*^52^ developed the injection-molded plastic array 3D culture (IMPACT) platform, which simplifies and diversifies the patterning of vascular structures within 3D cellular hydrogels.^49^ In this system, human umbilical endothelial cells (HUVECs) are seeded into the outer channel containing fibrinogen–thrombin hydrogel, where capillary forces facilitate droplet patterning along the channel. Lung tumor cells, such as A549, are introduced into the center channel in the same hydrogel, creating spatially organized vascular and tumor cell lumens. This arrangement promotes crosstalk between cell types and enables systematic drug response studies. The choice of hydrogel is critical for ensuring healthy cell behavior and providing appropriate mechanical properties for compartmentalized cell packing. Commonly used hydrogels include collagen I, fibrinogen, fibrin, and Matrigel, as reviewed in prior studies.^53,54^

The microwell platform is typically well suited for high-throughput applications and is often integrated with high-content screening tools to evaluate multiple drug treatment conditions on tumor cells. Its primary use is the generation of uniform tumor spheroids or organoids with a small number of cells, which requires careful optimization of the cell culture substrate material and microwell size.^55,56^ The combination of microwells and fluidic channel allowed the involvement of more cell types and environmental factors, such as the perfusion of immune cells, co-culture with vascular cells, and drug administration.^57,58^

The microchannel platform, utilizing microscale gradient generators (uGGs), creates precise biochemical gradients with controlled spatiotemporal distribution and subcellular resolution. uGGs rely on diffusive mixing between parallel laminar streams of different compositions to generate molecular gradients. To address shear stress issues caused by laminar flow, which can alter cell phenotypes and wash away secreted factors, advanced designs incorporate flow resistive elements or flow barriers like hydrogels to achieve passive diffusion. The uGGs are the perfect platform for drug sensitivity testing by generating different concentrations and combination of drugs in a time and reagent efficient manner. Xu *et al.* integrate the gradient concentration generator with 3D co-culture system of lung fibroblasts and NSCLC cell line (SPCA-1) or primary tumor cells, evaluating the drug response to gefitinib, PTX, and GCB simultaneously.^59^ Zhang *et al.* incorporated the automated discrete drug logarithmic concentration generation with cell culture chamber on chip to assess potency across different concentrations.^60^

The integration of diverse spatial designs representing tumor microenvironment (TME) architectures within microfluidic chips has unlocked significant potential for studying various tumor mechanisms and evaluating therapeutic efficacy. Advance in microfluidic chip design has increasingly incorporated cutting-edge biological technologies, such as microbead-based immunofluorescence,^61^ to enable precise molecular profiling. Additionally, the inclusion of electronic automation systems^62^ has enhanced the functionality and efficiency of these platforms, paving the way for more sophisticated and high-throughput investigations. In the next two sections, we will discuss the state of the art on microfluidic systems mimicking the complexity of normal lung microenvironment (lung-on-a-chip) and tumor microenvironment (lung cancer-on-a-chip).

## IV. LUNG-ON-A-CHIP: THE STATE OF THE ART

### A. The architecture of human lung

The lung, as the primary organ of the respiratory system, comprises various tissues and cell types that enable gas exchange between the bloodstream and the external environment. It consists of two main regions: the conducting portion and the respiratory portion. The conducting portion, including the bronchi and bronchioles, channels air to the respiratory portion, which begins at the alveolar ducts and culminates in the alveolar sacs and alveoli, the primary sites of gas exchange.^63^ The main cell types in the conducting portion of airway include basal cells, goblet cells, ciliated cells, and pulmonary neuroendocrine cells (PNECs) that control the inhaled particles and microbes in the airways, and the basal cells are the stem cells which can differentiate into the other three cell types.^64^ In the respiratory portion, the alveolar type II (AT2) cells produce surfactant proteins and have the progenitor features to differentiate into alveolar type I (AT1) cells, which facilitate gas exchange with the surrounding capillaries.^64^ In adult lung, the homeostasis and regeneration of epithelium are supported by several mesenchymal cell types, such as airway smooth muscle cells, vascular smooth muscle cells, chondrocytes, and alveolar fibroblasts.^64^ Additionally, lung endothelial cells (ECs) show region-specific specialization. In the alveolar capillaries, two major endothelial subtypes coexist: aerocyte capillary (aCap) cells, which interface closely with AT1 cells to optimize gas exchange, and general capillary (gCap) cells, which serve as progenitors for aCap cells.^65^ These capillaries also encounter complex mechanical tensions due to dynamic changes in airflow, blood flow, and alveolar expansion during respiration, which can be readily modeled in microfluidic systems given their high spatiotemporal control capability.

Regular exposure to harmful stimuli, such as pathogens, air pollutants, and cigarette smoke, frequently damages the lung epithelium, challenging its self-repair mechanisms and increasing the risk of carcinogenesis. To preserve homeostasis, multiple stem and progenitor cells in the lung play critical roles in cellular and tissue regeneration. Notably, it is hypothesized that lung carcinogenesis exploits these stem cell repair pathways.^66^ Lung cancer is believed to originate from the transformation of progenitor cells harboring genetic mutations, which proliferate and evolve in cell identity and lineage, adapting to the lung's microenvironment over time.^66^

Lung cancer subtypes arise from different locations in the lung, indicating the diverse lung epithelial cell origins for tumor initiation.^67^ SCLC, for instance, arises in the central airways and expresses markers such as calcitonin gene-related peptide (CGRP), typically associated with PNECs, the progenitors of club and ciliated cells.^68,69^ Lung squamous cell carcinoma (LSCC), a subtype of NSCLC, commonly develops in the ducts of submucosal glands or at the tracheal and upper airway boundaries. It is characterized by a basal cell phenotype and the expression of markers like cytokeratin 5, P63, and SOX2, indicating its basal cell origin.^70^ Lung adenocarcinoma (LUAD), the most prevalent lung cancer type, is frequently found in distal airways and is believed to originate from AT2 cells.^63,66^ These cells are highly susceptible to oncogenic insults, as evidenced by their rapid proliferation under the activation of EGFR and KRAS signaling pathways.^71^ Additionally, AT2-originated LUAD cells exhibit plasticity, with reports showing transformation into highly proliferative AT1-like cells following KRAS inhibition, underscoring their role in drug resistance.^72^

**FIG. 3.**
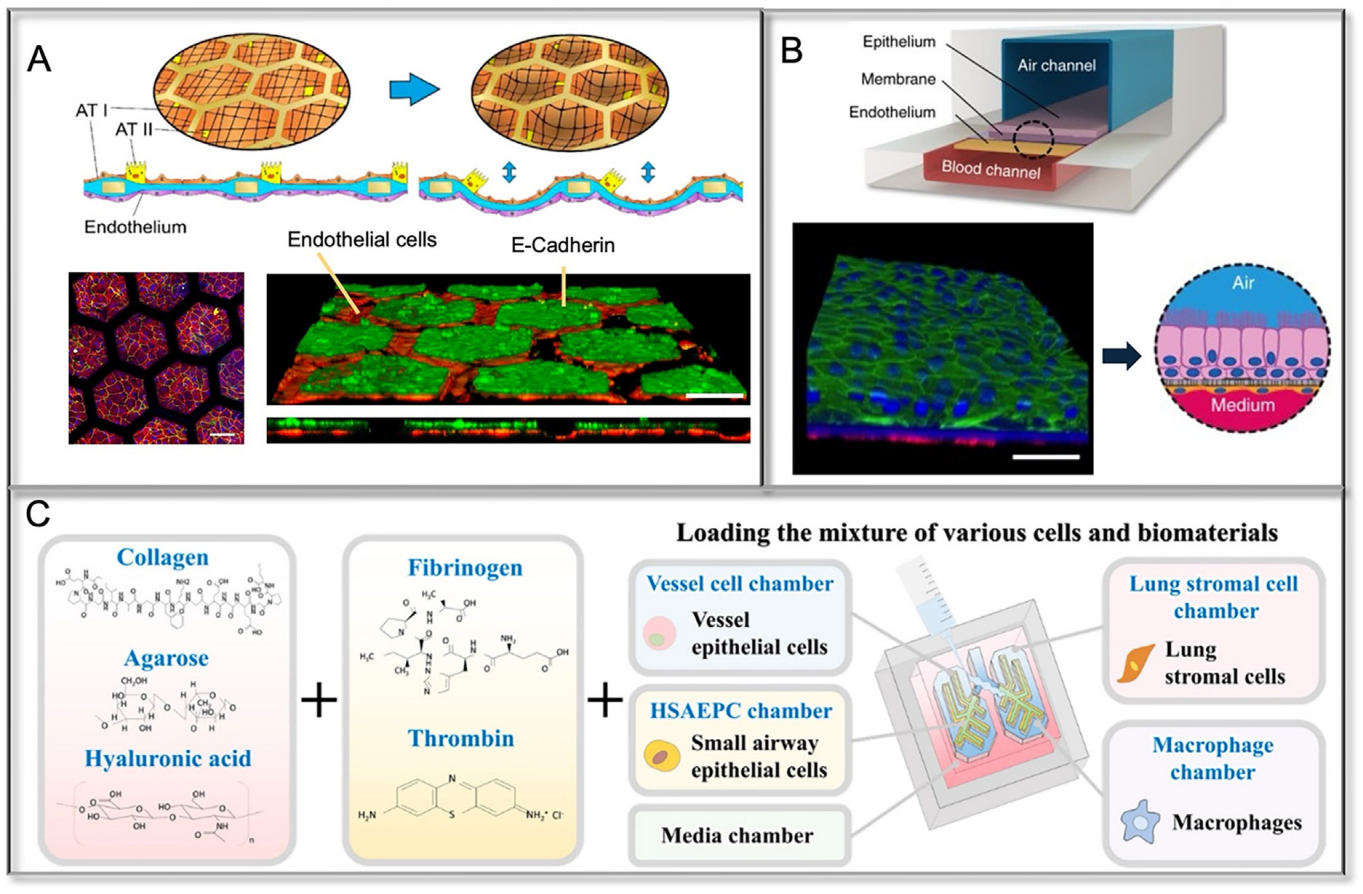
Microfluidic lung-on-a-chip models. (a) Type I (AT1) and type II (AT2) primary human lung alveolar epithelial cells are co-cultured with lung endothelial cells on the thin collagen–elastin membrane. Reproduced with permission from Zamprogno *et al.*, Commun. Biol. **4**(1), 168 (2021). Copyright 2021 Author(s), licensed under a CC-BY 4.0.^77^ (b) Diagram of a cross-section through airway-on-a-chip. The differentiated airway epithelium (pink cells) and the endothelium are at opposite sides of an air–liquid interface membrane. Reproduced with permission from Benam *et al.,* Nat. Methods **13**(2), 151 (2016).^80^ Copyright 2016 Springer Nature America, Inc. (c) A lung-on-a-chip platform was designed to replicate the microenvironment of the lung tissue and facilitate multicellular communication between the respiratory airways and adjacent supporting stromal chambers. Reproduced with permission from Min *et al.,* Int. J. Biol. Sci. **21**(1), 17–39 (2025). Copyright 2025 Author(s), licensed under a CC-BY 4.0.^81^

Given the heterogeneity of lung cancer subtypes and their cellular origins, accurately modeling the native physiological environment of normal lung tissue is essential for understanding the biological mechanisms driving tumorigenesis and progression. This includes replicating the composition of key cell types (e.g., lung epithelial cells, mesenchymal cells, and vascular cells) and designing appropriate culture conditions, such as extracellular matrices, cell–cell interaction, and mechanophysiological cues. This section summarizes the selection of lung cell types for designing lung-on-a-chip platforms (Fig. 3). It also explores the impact of various culture conditions, such as the choice of culture matrices and the architecture of cell–cell interactions, on effectively recapitulating the native lung microenvironment.

### B. 2D lung-on-a-chip models for physiological reconstruction

Most lung-on-a-chip microsystems utilize lung cell lines or primary cells **(**Table III**)** to recreate the lung microenvironment. Cell lines, such as BEAS-2B^73^ and 16HBE14o-,^74^ with their ease of acquisition and immortalized characteristics, ensure the availability of large quantities of consistent samples. However, their genetic modifications may lead to altered or diminished cellular physiological properties. To address this limitation, primary cells directly isolated from pulmonary tissue without artificial modifications are also widely incorporated into microfluidic systems.

**TABLE III.** Cell sources for lung cancer organoid models. HPMEC: human pulmonary microvascular endothelial cells; AEC: alveolar epithelial cells; CAF: cancer-associated fibroblast; HUVEC: human umbilical vein endothelial cells; PBMC: peripheral blood mononuclear cells; TIL: tumor-infiltrating lymphocytes; CTC: circulating tumor cells; ASC: adult stem cell; PSC: pluripotent stem cell; ESC: embryonic stem cells; PDO: patient-derived organoids.

Cell source	Examples	Advantages	Limitation
Cell line	A549, BEAS-2B71, 16HBE14o-, THP1	Easy and long-term maintenance; high reproducibility; high throughput; ease of genetic modification	Reduced physiological relevance; genetic, transcriptomic alteration; limited functional features
Primary cell	HPMEC, AEC, CAF, HUVEC, PBMC, TIL, CTC	Tissue-specific physiologic and functional properties; patient-specific disease modeling	Limited lifespan; donor variability; high cost; ethical and regulatory concerns
Stem cell	ASC, PSC, ESC	Self-renewal and differentiation; long-term usability; physiological relevance; patient-specific disease modeling	Complex culture conditions; batch and donor variability; immature differentiation; ethical and regulatory concerns
PDO	⋯	High physiological relevance; heterogeneous cell composition; personalized disease modeling; 3D architecture and microenvironment	High cost; complex culture conditions; lack of standardization; lack of vascular and immune system; longer differentiation time

The first microfluidic airway system was introduced by Huh *et al.* in 2007. This system employed primary human small airway epithelial cells (SAECs) to create an ALI chip, enabling precise replication of the physiological liquid plug flows observed in the respiratory system.^49^ This study revealed the injurious effects of liquid rupture-induced mechanical stress on lung epithelium. Notably, airflow interface in the ALI design significantly promoted SAEC differentiation and enhanced monolayer integrity under stress conditions compared to liquid culture. Later, Huh *et al.* developed a functional alveolar–capillary interface using human pulmonary microvascular endothelial cells (HPMECs) and primary alveolar epithelial cells (AECs) seeded on opposite sides of a porous membrane.^51^ This design mimicked breathing through membrane stretching and enabled studies on epithelial–endothelial interactions, neutrophil transmigration, and nanoparticle responses, advancing insights into lung physiology and pathology.

Unlike the distal alveoli, the airway mucosa comprises an epithelial surface, interstitial cells, and capillaries functioning as a unit. The thickness of the interstitial compartment decreases as bronchi become bronchioles, becoming a fraction of a micrometer in the alveoli that exert gas exchange. Thus, Sellgren *et al.* augmented the lung airway epithelial cell ALI cultures with primary lung fibroblasts and lung microvascular endothelial cells to pursue a greater physiologic complexity of the airway mucosa.^75^ To accommodate the co-culture of three mucosa cell types, they tested multiple combination of collagen I or IV as the coating substrate, which supported the differentiation of airway epithelium, as indicated by the presence of mucin granules and cilia. They carefully balanced epithelial (ALI1/2) and endothelial (EGM) media, addressing challenges such as FBS's dual role in fibroblast growth and epithelial differentiation. In addition to incorporating multiple lung airway cell types, the use of novel biomaterials has enabled the recreation of native lung biomechanics, thereby supporting the maintenance of cell type-specific identities and functions. Stucki *et al.* advanced alveolar–capillary modeling by successfully culturing primary AT1 and AT2 cells on a cyclic stretchable PDMS membrane for at least seven days.^76^ The deflection of this thin membrane was regulated through a passive mechanism driven by hydrostatic and surface tension forces. Building on this, Zamprogno *et al.* transitioned from traditional PDMS-based single-barrier alveolar models to a bioengineered alveolar array incorporating physiological extracellular matrix (ECM) components and mechanics. The collagen–elastin membrane, with its native geometry and tunable stiffness, more accurately recapitulated the physiological lung microenvironment and offered potential for modeling various lung pathologies. Furthermore, applying cyclic physiological stretch on chip promoted cell specification, with markers like Caveolin-1 (AT1 marker), SP-C (AT2 marker), and ZO-1 (tight junction marker) confirming maturation and epithelial function^76,77^ [Fig. 3(a)].

Co-cultures of airway epithelial cells and smooth muscle cells (SMCs), essential for tracheal structure, were also developed using collagen–Matrigel mixtures for adhesion and maintenance.^78^ Further complexity was achieved by integrating immune components into an ALI lung-chip system. Perfusing human whole blood into the chip's bottom channel enabled real-time thrombus formation analysis under inflammatory stimuli like TNF-α and LPS, highlighting increased platelet–endothelial binding^79,80^ [Fig. 3(b)]. Most recently, a lung-on-a-chip model was developed that incorporates multiple lung cell types, including lung stromal cells, vascular endothelial cells, lung epithelial cells, and macrophages, within a microstructure designed to mimic the respiratory compartments of the lung. The spindle-shaped respiratory airway chamber is lined with human small airway epithelial cells and surrounded by adjacent stromal chambers housing human stromal cells and macrophages, effectively creating a comprehensive representation of the lung tissue microenvironment^81^ [Fig. 3(c)]. These advancements enhance the physiological relevance of lung-chip models for studying airway and alveolar biology.

Overall, the development of primary cell and cell line-based lung chips has led to increased cellular complexity in the gas–fluid exchange unit and the incorporation of breath-mimicking mechanical forces. These lung chips have shown significant potential in modeling various respiratory diseases and pathological processes (e.g., asthma, thrombosis, viral infections),^79,80,82^ highlighting their translational value. By replicating the alveolar–capillary barrier, these 2D lung chips provide a physiological foundation for studying the lung cancer microenvironment, offering new insights into cancer initiation and progression.

### C. 3D organoid-based lung-on-a-chip models for physiological reconstruction

Although the 2D ALI microfluidic chip is a common method for modeling pulmonary gas-blood exchange, the primary cells are hard to be propagated as stable phenotypes in 2D cell culture. Thus, the 3D spherical organoid model with stem cell-derived lung epithelium is emerging as another *in vitro* method to study lung regeneration and cell–cell interaction. Organoids are self-organized structures composed of multiple cell types and extracellular matrix (ECM), mimicking the cellular composition and functionality of *in vivo* tissues. Unlike the ALI model, where primary cells are seeded into the system, organoids are derived from adult stem cells (ASCs) or pluripotent stem cells (PSCs).^83^

The lung ASCs are undifferentiated cells residing lung airway epithelial niche, including basal cells (NGFR+ITGA6+) in proximal airway and AT2 (HTII-280+) cells in the distal lung, giving rise to ciliate cells and AT1 cells, respectively.^84,85^ ASCs are quiescence during normal condition; however, when facing lung injuries, they can self-renew and differentiate into mature airway epithelial cells. These cells can be FACS-isolated from patient tissue and cultured in 3D matrices like Matrigel or collagen, allowing for the generation of organoids that maintain genetic stability and differentiate into specific epithelial cell types.^85^ On the other hand, PSCs, such as embryonic stem cells (ESCs) and induced pluripotent stem cells (iPSCs), can self-renew and differentiate into any cell type in the body. The development of lung organoid from PSCs is a complicated process involving multiple growth factors or small molecular in differentiation medium to recapitulate the signaling pathways that regulate *in vivo* organ development.^86^ The immortalized culture of iPSCs enables large-scale organoid production and the potential for genetic modifications in specific cell types.

Directed differentiation, an *in vitro* method to mimic early embryo development by activating or inhibiting specific signaling pathways, has been used in PSC-derived organoid studies to generate lung tissue lineages. By inducing definitive endoderm through Activin, inhibiting TGF-β/BMP pathways, and activating BMP/FGF signaling, a population of Nkx2-1+ respiratory progenitors can be generated and differentiated into lung epithelium.^87^ Although strategies to generate lung-specified endoderm are well-established, the induction of mature lung lineages remains under investigation.

Recent advances have shown that 3D cultures of lung progenitors in hydrogel, such as Matrigel, can produce mature AT2 cells expressing SFTPC.^86,88^ Currently, AT2 cells can be cultured as spheroids in Matrigel, while airway progenitors form epithelial spheres containing ciliated and secretory cells.^89^ A key factor in efficiently generating specific lung lineages is the temporal regulation of signaling pathways. For instance, BMP signaling must transition from inhibition during AFE specification to activation to promote lung development.^90^ Inhibition of Gsk3b using CHIR9902 is essential for generating distal lung progenitors and AT2 cell spheroids, while its removal facilitates differentiation into both proximal and distal lung cells, including AT1 and AT2 cells and basal cells.^89,91^ In addition, maintaining high purity in lung progenitor populations is crucial. Reporter constructs targeting genes like NKX2.1, SFTPC, or SCGB3A2 help purify specific lung cell types via flow cytometry, though these methods are not suitable for clinical applications.^91–93^ Alternatively, surface markers such as carboxypeptidase M or CD47^hi^/CD26^lo^ cells can effectively enrich lung endoderm progenitors for therapeutic purpose.^93,94^ Optimizing protocols to generate PSC-derived whole lung organoids containing both distal and proximal epithelial cells, along with mesenchymal cells, offers a more comprehensive platform for studying lung epithelial function in various pathological contexts and evaluating drug cytotoxicity. Moreover, the methods for the integration of vascular structure and immune components in lung organoid is the future direction for lung-on-a-chip.^95^

## V. LUNG CANCER-ON-A-CHIP: THE STATE OF THE ART

### A. Modeling tumor microenvironment on chip

Despite treatment advances in surgery, radiation, and targeted therapies, the prognosis of lung cancer remains poor due to the locally advanced and metastatic tumors in many patients at the time of diagnosis. The genomic landscape of NSCLC has been well defined in terms of the molecular subtypes. For example, mutations in *EGFR*, *KRAS*, *ROS1*, and *ALK* are considered oncogene-addicted and therefore sensitive to targeted therapies. However, the response rate and drug efficacy in most patients are limited by heterogeneous resistance mechanisms.^3,96^ Accurately identifying lung cancer subtypes and predicting their treatment responses can support personalized therapy design at early stages. Moreover, close monitoring of tumor progression during treatment is essential for timely adjustment of therapy, given the inevitable emergence of resistance.

It is well accepted that the TME plays a critical role in the initiation and progression of primary lung carcinoma, and the enrichment of cell types in TME provides more targets for the development of anticancer agents.^96^ The distinct cellular heterogeneity of lung cancer spans the both tumor epithelial cells and TME, including vasculature, cancer-associated fibroblasts (CAFs), ECM, and infiltrating immune cells. At the organ level, the chronic inflammatory state in the lung displays features contributed to the carcinogenesis, indicating the lung tissue homeostasis in affecting tumor initiation.^97^ Enrichment of CAFs correlated with poor prognosis that associated with metastasis.^98^ Increased tumor-infiltrating lymphocytes (TILs) is associated with better prognosis and higher survival,^99^ while regulatory T cells were associated with worse recurrence-free survival.^100^ Given the strong influence of TME on lung tumor progression, appropriate models that mimic the *in vivo* TME features are fundamental to finding new therapeutic targets and testing efficacy.

**FIG. 4.**
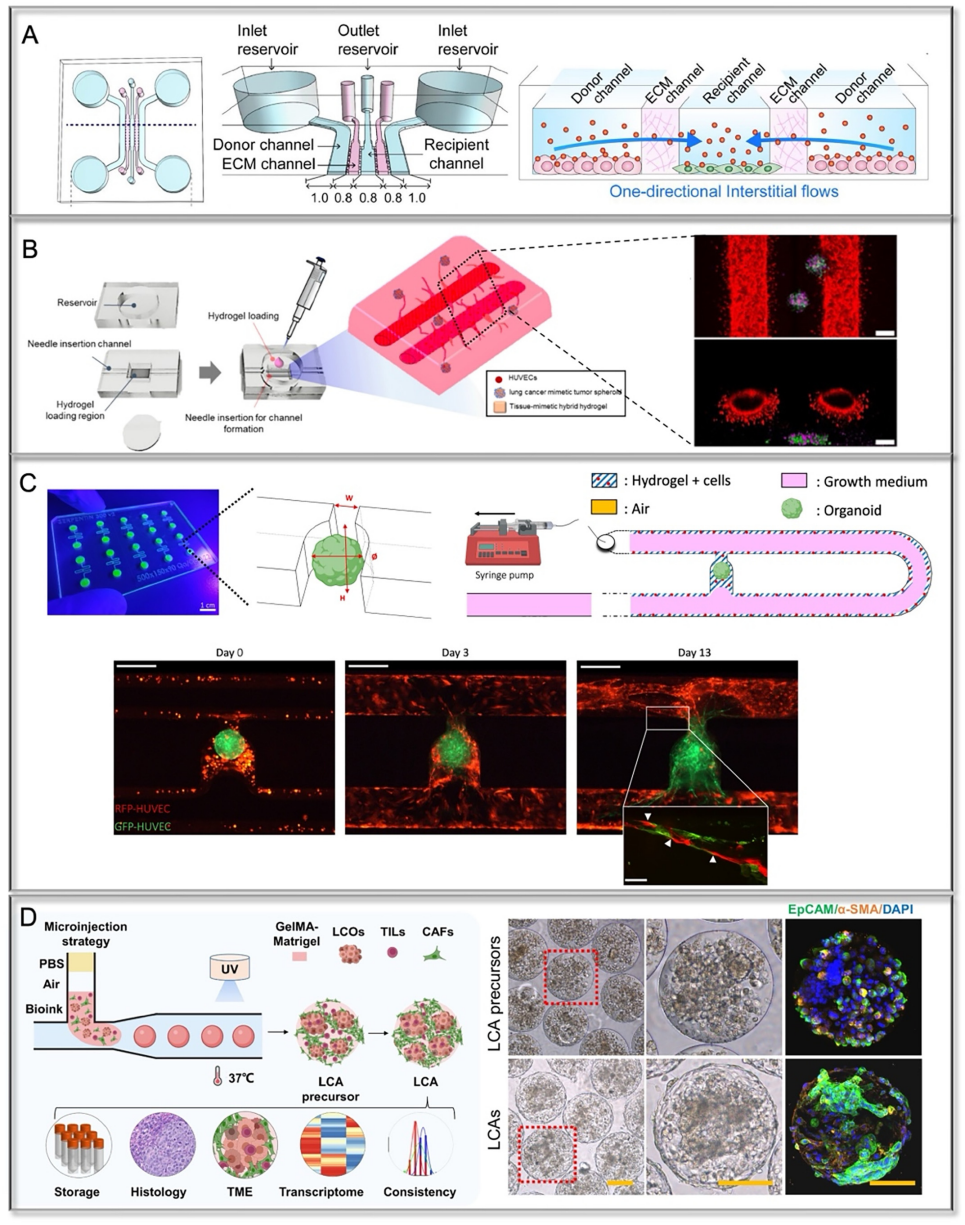
Multicellular lung cancer-on-a-chip systems. (a) Co-culture of fibroblasts with lung cancer cells in one-directional flow chip for studying paracrine signaling between cancer cells and fibroblasts. Reproduced with permission from Kim *et al.,* Acta Biomater. **144**, 258–265 (2022).^108^ Copyright 2022 Elsevier. (b) A vascularized lung cancer-on-a-chip platform using sacrificed vascular channels with tumor spheroids encapsulated in the hydrogel. Reproduced with permission from Park *et al.,* Cancers **13**(16), 3930 (2021). Copyright 2021 Author(s), licensed under a CC-BY 4.0.^113^ (c) A functional vascularized microfluidic chip with U-cup-shaped area functioning as a trap for growing organoid. Growth medium was introduced for continuous perfusion of the microfluidic chamber and the trapped organoid. Reproduced with permission from Quintard *et al.,* Nat. Commun. **15**(1), 1452 (2024). Copyright 2024 Author(s), licensed under a CC-BY 4.0.^114^ (d) Patient-derived lung cancer assembloids generated using microinjection-based cell-laden hydrogel manipulation to reserve multiple cell types from *in vivo*. Reproduced with permission from Zhang *et al.,* Nat. Commun. **15**(1), 3382 (2024). Copyright 2024 Author(s), licensed under a CC-BY 4.0.^118^

To this end, the microfluidic platform has been emerging as a multifunctional *in vitro* lung cancer modeling system. It enables the co-culturing of multiple TME cell types in a controllable spatial organization with specific ECM properties that mimic the tumor tissue geometry (Fig. 4). The incorporation of engineering factors, such as fluidic flow, air–liquid interface, interstitial pressure, oxygen gradient, and biosensors, further benefits the application of lung cancer-on-a-chip in lung tumor diagnosis, cancer stage monitoring, and drug testing.

### B. Cellular complexity on lung cancer-on-a-chip

#### 1. 3D modeling of lung cancer-on-a-chip

Cancer cell lines (Table III) have long served as the primary tool for investigating biological phenotypes due to their easy acquisition, ability to expand, and consistent passages. The US National Cancer Institute has established 60 human tumor cell lines, including 9 NSCLC lines, for drug screening.^101^ These cell lines are extensively characterized at various molecular levels, such as DNA methylation, mutation burden, RNA expression, and metabolic activity, providing a solid foundation for *in vitro* mechanistic studies. Additional lung cancer cell lines, such as NCI-H1975 (*EGFR*^T790M^) and NCI-H358 (*KRAS*^G12C^), are valuable for exploring drug resistance mechanisms.^102^ Over recent decades, 3D tumor spheroid cultures have gained attention for more accurately replicating tumor structures, including cell shape and density. Tumor spheroids can form either through spontaneous aggregation or by embedding cells in hydrogels like Matrigel or collagen. While these spheroids may not fully mimic the histological pattern of the original tumor, they do replicate key metabolic and proliferation gradients, making them useful for high-throughput screening.^103^ However, cancer cell lines alone cannot completely capture the complexity of *in vivo* tumors. The primary cancer cells dissociated from tumor tissue serve as an alternative for *in vitro* modeling. It was reported that freshly dissociated lung tumor cells from patient samples could form spheroid by embedding into IrECM derived from the Engelbreth–Holm–Swarm mouse sarcoma cells.^104^ Patient-derived organoids (PDOs), which are directly obtained from surgical samples or biopsies, preserve the native tumor histology and are increasingly viewed as ideal models.^103^ Table III summarizes the common cellular sources for modeling lung cancer, and Fig. 2 shows common sample types (tissue, blood, pleural effusion, and bronchoalveolar lavage) that may be collected during the course of lung cancer disease management.

Tools like hydrogel embedding and ultra-low attachment plates are commonly used for spheroid formation or PDO subculture, though they are cell-type dependent, challenging to control morphologically, and labor-intensive. Recent improvements in microwell chips with agarose coatings have enabled consistent spheroid and PDO formation, facilitating high-throughput screening and drug testing.^55,105^ The low cost of agarose and the efficiency of these chips have made them highly valuable. However, the limited size of tissue biopsies and PDOs presents challenges for their seeding and expansion. Tissue trapping chips, designed to efficiently trap individual biopsies^106^ or PDOs,^57^ have been developed to test chemotherapy efficacy. Recent PDO trapping chip has also been used to monitor interactions between PDOs and autologous immune cells, evaluating lymphocyte-mediated killing behavior.^61^

#### 2. Multicellular organoid models with CAFs

Given the crucial role of the TME in tumor progression, drug response, and resistance, reconstituting *in vitro* TME models is an emerging trend in the field. CAF, one of the most abundant and heterogeneous cell types within the TME, significantly influence tumor behavior. The presence of tumor-like CAFs is strongly associated with poor prognosis. Different CAF subtypes play different functions in TME and the associated drug response.^107^ The inflammatory CAFs and interferon-response CAFs are associated with inflamed TME, indicating the good prognosis, while the ECM-producing CAFs are correlated with low immune infiltration and poor prognosis.^107^ It is noteworthy to recapitulate the cellular interaction between CAFs and tumor cells in lung cancer-on-a-chip model.

An early study mixed lung cancer cell line SPCA-1 with human lung fibroblast cell line HFL1 in Geltrex and injected in chip coupled with fluidic flow.^59^ The concentration gradient generator on chip enabled simultaneous testing of various drug concentration in tumor–fibroblasts chambers, screening the most sensitive treatment condition. The paracrine interaction between tumor cells and fibroblasts was investigated in the microfluidic channels, where the tumor cells and lung fibroblasts were separated by collagen I filled channel^108^ [Fig. 4(a)]. In another study, the head pressure between channels generated the unidirectional flow from tumor cells to fibroblasts, facilitating the delivery of secretory factors. This paracrine crosstalk reshaped the normal lung fibroblasts into CAF-like phenotype, including increased migration and invasion accompanied by upregulation of CAF activation markers (e.g., *MMP-9*, *FAP*, *Vimentin*). However, due to the difficulty in isolating primary CAFs and the challenges of long-term culture-induced senescence, the use of primary CAFs in cancer-on-chip studies is limited.^109^ Tumor cell-induced transformation of normal fibroblasts into CAF-like cells offers a promising alternative for recreating the TME.

#### 3. Multicellular organoid models with vascular cells

The blood vessels, composed of an inner endothelial layer and outer pericytes, play a crucial role in the TME, influencing tumor aggressiveness and drug response. Tumor blood vessel is disorganized, leaky, and tortuous, with poor pericytes or smooth muscle cells coverage.^110^ The collapsed, narrowed lumen of tumor vessels leads to low perfusion and a hypoxic environment, hindering immune cell infiltration and drug delivery.^111^ Crosstalk between tumor cells and blood vessels also facilitates metastasis, where local tumor cells transition to a mesenchymal state and invade blood vessels through the endothelium. Once in the bloodstream, tumor cells can spread and seed distant organs. However, the low fraction of endothelial cells (ECs) within the TME makes isolating primary tumor vessel ECs difficult. As a result, human umbilical endothelial cells (HUVECs) are commonly used in tumor-on-chip systems, although they are not tumor-derived. Some studies have also used iPSC-derived ECs as an alternative.^78^ The advantages of iPSC-ECs in tumor-on-chip models include their immortality and potential to adopt TME-associated phenotypes when co-cultured with tumor cells.

**FIG. 5.**
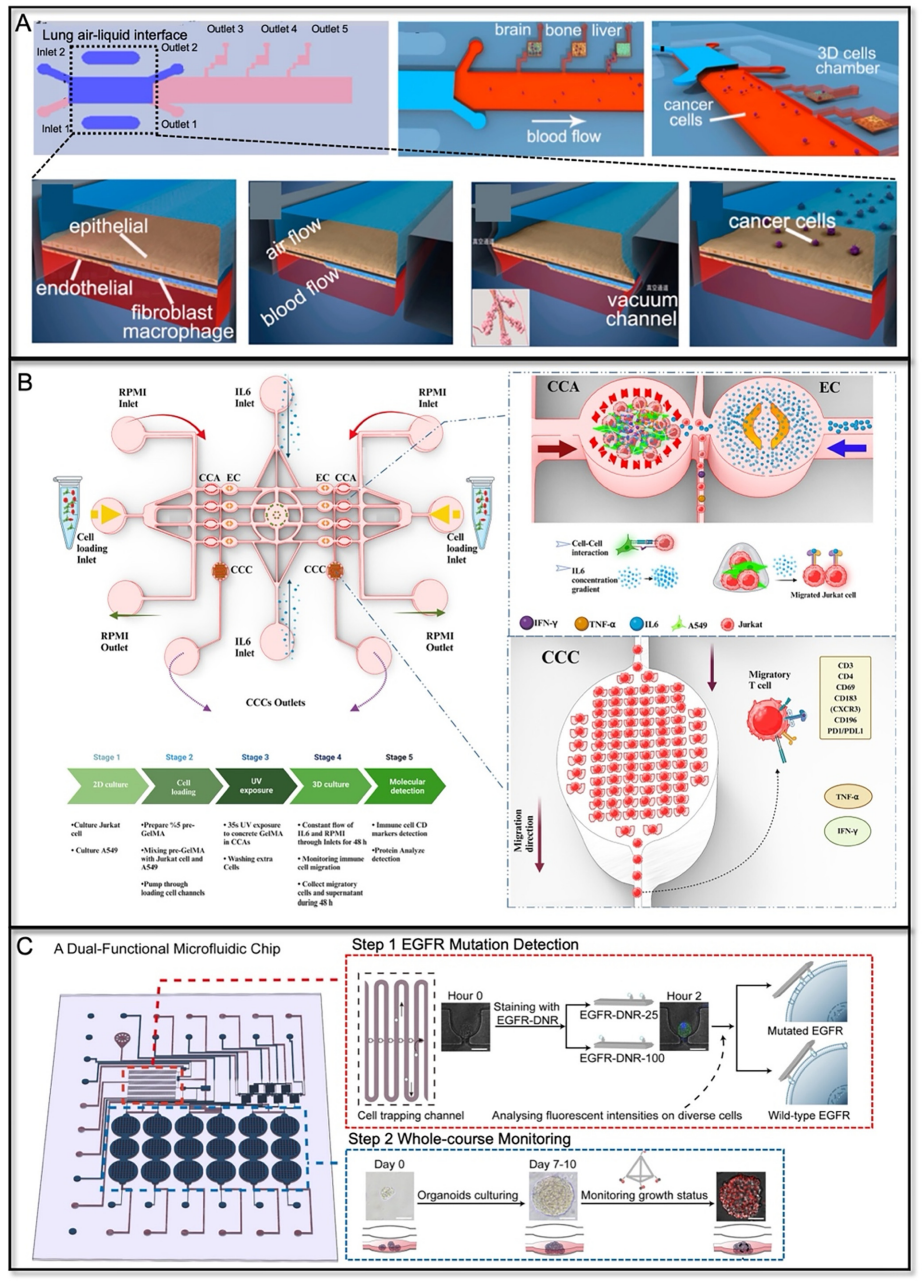
Lung cancer-on-a-chip application in diverse therapeutic modalities. (a) A multiorgan microfluidic chip that includes an upstream “lung organ” and three downstream “distant organs.” Reproduced with permission from Xu *et al.,* ACS Appl. Mater. Interfaces **8**(39), 25840–25847 (2016).^112^ Copyright (2016) American Chemical Society. (b) A symmetric flow-based lung cancer-on-chip model for studying T-cell activity in response to interactions with lung cancer cells and varying IL-6 gradient concentrations. Reproduced with permission from Sardarabadi *et al.,* ACS Appl. Mater Interfaces **17**, 4354 (2025). Copyright 2025 Author(s), licensed under a CC-BY 4.0.^119^ (c) Rapid EGFR mutation detection and drug response test in a dual-functional microfluidic chip. Adapted with permission from Zhang *et al.,* Lab Chip **24**(6), 1762–1774 (2024).^134^ Copyright 2024 Royal Society of Chemistry.

In one early study, A549 cells and lung epithelial cell line 16HBE were co-cultured on the upper side of a membrane, while the HUVECs and macrophages were on the opposite side. The lower blood vessel channel was perfused and connected to several portals cultured with typical cell types from brain, liver, and bone. The migration of tumor cells through endothelium toward downstream organ-like portals could be monitored at the single-cell level, mimicking the metastasis^112^ [Fig. 5(a)]. However, this kind of microchannel chip could not reflect the spontaneous assembly of vessel structure seen in tumor and the cell crosstalk was restricted by the membrane. Lee *et al.* improved this approach by seeding collagen-embedded A549 cells in microwells connected to a microfluidic channel, with HUVECs forming an endothelial layer at the junction between the channel and microwells. The fluidic flow facilitated the integrity of the endothelial layer and promoted angiogenesis of ECs into tumor cell aggregates in each well.^58^ A similar “lumen chip” design created endothelial channel and incorporated tumor-lung fibroblast clusters in decellularized porcine lung-derived hydrogel. The tissue-mimetic hybrid hydrogel and the existence of fibroblasts promoted the angiogenesis of tumor spheroid, and it allowed for perfusion in endothelial channel to facilitate the drug screening^113^ [Fig. 4(b)]. This model successfully recapitulated tumor–stroma–vasculature interactions and demonstrated drug responses, though the vascular complexity was limited to large endothelialized channels with short-term culture. More recently, a study cultured iPSC-derived vessel organoids on a chip, enabling the formation and spread of perfused microvascular networks, providing a new resource for vascularizing 3D tumor tissues^114^ [Fig. 4(c)]. This versatile microfluidic platform enabled self-assembly of perusable microvascular networks within hydrogel-embedded organoids, maintaining long-term perfusion and vessel functionality. Unlike primary endothelial cells such as HUVECs, iPSC-derived endothelial cells possess a more naïve cellular identity, offering the potential to be further directed or “trained” toward phenotypes that more closely resemble the microvascular characteristics of the lung TME.

Overall, fluidic flow, matrix composition, and multicellular interactions (e.g., fibroblasts) are critical in influencing vascular structure formation and growth on chips.^115^ Additionally, tissue clearance and imaging techniques should be integrated with microfluidic systems for comprehensive and quantitative analysis of vascular networks.

#### 4. Multicellular organoid models with immune cells

There are various immune cells reside in TME, including lymphocytes, tumor-associated macrophages (TAMs), natural killer cells, dendritic cells, and cancer-associated neutrophils. These immune cells play complex roles in regulating the tumor response to treatment.^45,116^ Immunotherapy, including immune checkpoint inhibitors, has significantly improved treatment outcomes for advanced-stage NSCLC. Incorporating immune cells into microfluidic systems is essential for recreating a close-to-actual TME and studying tumor responses to immunotherapy on a chip.

To recapitulate the native TME, THP1, a monocyte cell line, was mixed with tumor cells and fibroblasts as spheroids.^117^ It was observed that THP1 could be transformed into CD68+ TAMs after the long-term co-culture with tumors. However, since immune cell lines still cannot reveal the features of tumor-specific immune cells, the use of autologous cells from patients, such as tumor-infiltrating lymphocytes (TILs), offers a more authentic representation of the TME. A recent study used droplet microfluidic technology to assemble patient-derived TME cells, including CAFs and TILs, with PDOs in the GelMA-Matrigel microgels^118^ [Fig. 4(d)]. The generated tumor assembloid model demonstrated good intrabatch consistency in size, cell composition, and drug response profiling.

Other than directly incorporating immune cells with tumor cells, the perfusion of lymphocytes in microchannels on chip can benefit the investigation of immune activation in the TME. One study designed ideal chambers and channels to form a concentration gradient generator chip, mimicking T cells interaction with cancer cells in the presence of different concentration gradients of IL6 in TME.^119^ This study emphasizes the dual effects of IL-6 on T-cell activation, exhaustion, and cytokine production, providing valuable insights into immune response modulation. Given the heterogeneous and phenotypic plasticity of tumor-associated immune cells, it is necessary to achieve the effective monitor and quantitative analysis of immune cells activities on chip (e.g., migration, proliferation, and activation) via imaging and secretory profiling.^61^

#### 5. Circulating tumor cell (CTC) studies

In addition to tumor cells from solid tumor biopsy, the collection and analysis of circulating tumor cells (CTCs) from liquid biopsy have emerged as an effective and non-invasive way to support early detection, monitoring response to treatment, and predicting disease recurrence. These cells hold the potential of supplying the cell source for real-time modeling of lung cancer *in vitro*. CTCs are shed from the primary tumor and circulate in the blood, and they are suggested to be the potential founders of distant metastasis.^120^ Due to their low numbers in circulation, advanced techniques are required to accurately identify and isolate CTCs from limited blood samples. The CellSearch system, an immunomagnetic enrichment based on EpCAM expression, is the only FDA-approved method for CTC detection. However, this system dismisses the potential CTC subtypes that are negative for EpCAM. An alternative method involves size-dependent microfluidic enrichment, taking advantage of the relatively larger size of CTCs compared to blood cells.^121,122^

During the course of treatment, the molecular landscape of tumor cells undergoes continuous evolution, ultimately leading to the development of acquired resistance to therapy.^123^ Lung cancer models from CTCs, including CTC-derived organoids,^124^ offer a minimally invasive approach to monitor tumor dynamics and can provide timely insights to guide therapeutic modifications, as CTCs can be obtained from patient blood samples without causing harm. *In vitro* culture and characterization of CTCs offer opportunities to explore drug susceptibility patterns in individual patients, aiding in the development of tailored therapeutic strategies. Several culture methods for CTCs were reported, including the non-adherent culture condition,^125^ microfluidic fibroblast co-culture model,^126^ and white blood cell co-culture model.^127^ Using microfluidic platform and proper formula of culture conditions, precise enrichment and quick expansion of CTCs become promising. The phenotype analysis and drug testing of CTC-derived tumor organoids represent a new direction for personalized cancer therapy. Nevertheless, challenges such as low success rate in deriving organoids remain to overcome.^128^

### C. Commercialized lung cancer-on-a-chip platforms

Built upon decades of academic research and iterative design, lung cancer-on-a-chip platforms have increasingly been commercialized, offering standardized, reproducible, and user-friendly models that accelerate translational research and preclinical drug testing. Several commercial systems now integrate features that mimic key aspects of lung physiology. For example, the Chip-S1 by Emulate and the ^AX^Lung-on-chip by AlveoliX incorporate stretchable membranes to replicate the mechanical forces stemming from breathing. Similarly, the UBeat^®^ Stretch chip by BiomimX uses an actuation layer underneath the cell culture layer to transfer a controlled mechanical stimulation to the cell constructs. To recapitulate circulatory dynamics, platforms such as the Pod module of Chip-S1, MesoBioTech's organ-on-chip, and ComPLATE by Bi/ond include perfusable channels and flow control systems that extend tissue viability and support long-term modeling.

Beyond physiological relevance, many commercial platforms are tailored for specific research applications. The OrganoPlate by MIMETAS combines microfluidic flow with multiplexed 3D culture wells, enabling high-throughput co-culture and tumor–immune interaction studies. Systems like Cherry Biotech's CUBIX and Bi/ond's ComPLATE further support real-time monitoring through live-cell imaging in controlled perfusion environments. While these commercial lung-on-a-chip platforms are far from recapitulation of human lung physiology and microenvironment, they bridge the gap between academic prototyping and scalable application, facilitating disease modeling with enhanced physiological relevance, enabling more precise and rapid drug screening, and supporting high-throughput, multiparametric readout analysis.

## VI. MICROFLUIDIC APPLICATION IN DIVERSE THERAPEUTIC MODALITIES

The advent of immunotherapy (e.g., PD-L1 inhibitors) and targeted therapy (e.g., TKIs) has significantly shifted the treatment paradigm for lung cancer. The development of microfluidic platforms has enhanced the modeling of TME in lung cancer, providing broad opportunities for rapid and sensitive testing of various therapeutic modalities (Fig. 5). This advancement accelerates progress in personalized medicine by enabling more tailored and effective treatment strategies.

Metastasis is an important tumor progression event, and its occurrence usually accompanies tumor cell phenotype switch and requires the adjustment of the therapeutic strategies. For this point, multiorgan microfluidic chip model offers a physiologically relevant *in vitro* model of lung cancer metastasis^112^ [Fig. 5(a)]. The device used layered PDMS microchannels and microporous membranes to spatially segregate and yet fluidically connect the compartments, enabling tumor cells to migrate from the lung region into the downstream “organ” chambers under flow. Rather than simple 2D culture, the organ-on-chip design permitted more realistic evaluation of anti-metastatic drug effects, cell–cell and organ–organ interactions, and therapeutic resistance in a controllable microenvironment.

To evaluate the efficacy of immunotherapy, such as immune checkpoint inhibitors (ICIs), a dynamic on-chip TME that supports interactions between immune cells and tumor cells is essential. Moore *et al.* developed a multiplexed microfluidic system termed EVIDENT (*in vitro* immuno-oncology dynamic environment for tumor biopsies), which enables real-time, high-resolution imaging of interactions between autologous TILs and tumor fragments, allowing for the assessment of various ICI treatments and tumor responses.^129^ The compatibility of the chip with microplate readers or high-content imaging system enhances its usability. To account for the impact of ECM barriers on immune cell-mediated cytotoxicity, an injection-molded plastic array culture device was designed to evaluate the killing efficiency of cytotoxic lymphocytes within a 3D microenvironment. This platform provides spatiotemporal analysis of lymphocyte and cancer cell behavior in ECM, revealing that while dense ECM strengthens the physical barrier, cytotoxic lymphocytes effectively kill cancer cells upon direct contact.^130^ In a more advanced microfluidic platform for immuno-oncology modeling, tumor cells were embedded within a 3D hydrogel matrix while microfluidic uGGs were integrated to generate cytokine gradients and sustain continuous perfusion. This configuration created a physiologically relevant dynamic environment that enabled the study of T-cell chemotaxis and differential cytokine exposure across the chip.^119^ The system also incorporated multiple downstream analytical modules, including real-time cell tracking, flow cytometry, and cytokine quantification, allowing comprehensive assessment of immune responses within the tumor microenvironment^119^ [Fig. 5(b)]. This design highlights a future direction for immuno-oncology therapeutic modeling—toward more multiparametric, high-throughput microfluidic platforms that enable integrated evaluation of complex tumor–immune interactions. As new immunotherapy modalities, including immune checkpoint blockade and adoptive T-cell transfer, continue to emerge, preclinical models capable of assessing their ability to overcome immune suppression within the tumor microenvironment become increasingly important.^131^ The use of microfluidic tumor immunity models offers significant potential for predicting the efficacy and clinical outcomes of personalized immunotherapies.^131^

The utility of microfluidic device for mutation detections (e.g., EGFR) and TKI drug testing on patient tumor samples can facilitate the personalized treatment strategy designing. Hassell *et al.* used H1975 cell line which carries *EGFR*^T790M^, the mutation driving resistance to TKIs, in the breathing mimicking ALI chip to model the orthotopic lung tumor growth and invasion. It was validated that the H1975 cells were more sensitive to the third generation TKI (rociletinib) than the first-generation TKI (erlotinib).^47^ More interestingly, the breathing motion significantly increased the drug resistance of H1975 cells to the inhibitory effects of rociletinib, providing more evidence of the drug resistance mechanism in NSCLC. Shigeto *et al.* utilized a combination of single-cell microarray chips and peptide nucleic acid (PNA)–DNA probes to separate H1975 cells, which harbor *EGFR*^T790M^ mutations, from non-mutated cancer cells (A549), using fluorescence signals for labeling.^132^ Shen *et al.* developed a digital microfluidics-based FISH (DMF-FISH) platform that automates the traditionally labor-intensive FISH procedure for detecting EGFR amplification in NSCLC.^133^ The system integrates droplet handling, temperature control, and signal processing, enabling efficient cell digestion, hybridization, and staining with minimal manual intervention.

Recently, a dual-functional microfluidic chip was developed to detect EGFR mutations in PDOs within 2 h using a DNA-based nanoruler. The mutation detection results guided PDO-based drug response testing for TKIs or chemotherapy. Additionally, a DNA-based nanosensor enabled real-time monitoring of ATP levels to assess PDO growth^134^ [Fig. 5(c)]. This integrated platform offers a personalized approach to lung cancer treatment by combining mutation detection and drug sensitivity testing. Currently, most lung cancer drug resistance studies use mutation-carrying tumor cell lines and focus on the detection of drug-resistant cells *in vitro*. However, the mechanisms by which new mutations are acquired after drug treatment are elusive and^134^ individually relevant. Microfluidic platforms offer an exciting opportunity to replicate the mutation acquisition process using patient-derived tumor cells cultured on-chip, enabling more personalized and effective drug resistance research and the identification of new therapeutic targets.

## VII. CONCLUDING REMARKS AND OUTLOOK

The microfluidic-based lung cancer-on-a-chip technology is advancing rapidly, demonstrating broad applications in the diagnosis and treatment of lung cancer. Compared to traditional 2D cell culture and 3D organoid models, lung cancer-on-a-chip systems recreate a miniature microenvironment that closely mimics the physiology of lung tissue and cancer. Features like ALI co-culture of lung epithelial and capillary endothelial cells emulate the gas-blood exchange unit, while 3D co-culture of tumor cells with stromal cells and perfusion channels replicates the TME for disease modeling and drug testing. These systems have significantly enhanced our understanding of lung tumor initiation, progression, and metastasis in a resource-efficient manner. However, challenges remain in fully replicating the lung cancer microenvironment at the tissue and organ levels, such as collection and subculturing of native tumor tissue, normal lung tissue–tumor interaction, and multiorgan microphysiological systems for metastasis and drug metabolism studies. Integrating lung cancer-on-a-chip platforms with technologies like high-throughput imaging, biosensors, and artificial intelligence (AI) could unlock new opportunities for clinical diagnostics and therapeutic development.

The adoption of PDOs over conventional cell lines is gaining traction in the organ-on-chip field due to their superior ability to recapitulate the *in vivo* tumor environment and enhance personalized medicine. However, significant challenges persist, particularly the variable PDO culture success rates, which range from 7% to 87% based on sample type and processing methods.^135^ Surgical resection specimens generally yield higher success rates compared to smaller volume samples, such as percutaneous needle or lymph node biopsies.^135^ Another limitation lies in the contamination of PDO cultures with normal lung mesenchymal cells, necessitating the optimization and standardization of key processes, including sample collection, digestion, medium formulation, and biophysical microenvironment design. Once established, the validation of lung cancer PDOs to ensure faithful representation of the original tumor is critical. Basic parameters, such as morphology and histological markers (e.g., thyroid transcription factor-1, cytokeratin 5/7, and p63), can be assessed through H&E staining and immunostaining.^136^ Advanced genetic and transcriptomic approaches, including whole-exome sequencing, copy number variation analysis, single-cell RNA sequencing, and spatial multiomics,^137,138^ provide deeper insights into tumor identity. Additionally, periodic validation of PDOs during long-term culture is essential to monitor potential cell population shifts across passages, ensuring the consistency and reliability of the model for downstream applications. CTCs have emerged as an alternative cell source for advancing disease monitoring and metastasis research. Key challenges in CTC analysis include achieving high purity during isolation, enabling efficient single-cell analysis, and optimizing the rapid expansion of CTC clones. Improvements in microfluidic-based CTC enrichment techniques, along with advancements in culture media composition and substrate design, hold significant potential to enhance the efficiency and applicability of CTC-based lung cancer research.^125,126^

Despite advancements in reconstructing the TME with cancer and stromal cells, current lung cancer organoid models still fall short of replicating the lung-specific physiological environment. Integrating tumor organoids with lung epithelial cells, capillary endothelial cells, and resident immune cells remains a key challenge. Primary lung epithelial cells and endothelial cells that used in most lung-on-a-chip model may not reveal the specific characteristics of AT1 and AT2 cells and their interaction with lung capillaries. Co-culturing stem cell-derived alveolar organoids with tumor organoids offers a promising approach to recreating the lung microenvironment, though efforts are needed to promote the maturation of alveolar structures, particularly alveolar type 1 (AT1) cells, which experience higher mechanical tension.^139^ Microfluidic platforms applying controlled mechanical forces could aid this maturation process. Another critical aspect is replicating the oxygen gradient between alveoli (∼13.3 kPa) and the hypoxic tumor microenvironment (<2.03–3.04 kPa).^140^ Well-designed gas channels on chips could facilitate hypoxia-targeted therapy studies. Furthermore, when co-culturing tumor and normal lung organoids, the functional identity of alveolar cells—such as marker expression, surfactant production, inflammatory signaling, and viability—must be closely monitored. This necessitates microfluidic chip designs compatible with staining, imaging, and immunoassay platforms to effectively evaluate tumor–lung cell interactions.

While most studies using patient-derived tumor materials focus on advanced-stage cancer, modeling early tumorigenesis on-chip represents a critical yet underexplored area. Genetic-engineered mouse models have demonstrated that the loss of *Rb1* and *p53* induces SCLC,^141^ while the mutation in *Kras^G12D^* and *p53* loss in AT2 cells could generate LUAD model in both mouse and human iPSC-derived lung organoids.^142^ Incorporating inducible mutations into cancer-prone lung cell types, such as basal cells for SCLC or AT2 cells for NSCLC, could help elucidate the transformation processes in early tumorigenesis. Microfluidic devices provide a platform for high-throughput and biophysically controllable propagation of mutation-carrying lung organoids, enabling long-term monitoring of early tumor cell transformation through cell-type-specific marker expression. At later stages, these systems can facilitate drug screening and response evaluation on emerging tumor cells, helping to identify valuable early therapeutic targets.

The integration of microfluidic platforms with other up-to-date technologies such as biosensors and artificial intelligence (AI) also holds immense potential for advancing lung cancer-on-a-chip research. Biosensors, capable of real-time monitoring of biochemical and biophysical signals, can be incorporated into microfluidic systems to dynamically measure critical parameters, such as oxygen gradients, cytokine secretion, and drug response biomarkers. While AI excels at analyzing complex and large volume datasets to identify patterns, predict outcomes, which help the optimization of experimental conditions, AI-driven image analysis could improve the detection of subtle morphological changes in 3D cultures and improve the output efficiency of personalized drug testing. By combining the high-throughput capabilities of microfluidic platforms with the precision of biosensors and the analytical power of AI, sophisticated lung cancer-on-a-chip models can be created to better replicate the complexity of lung cancer *in vivo*, accelerating drug discovery and improving therapeutic outcomes.

The integration of microfluidic devices into lung cancer research presents several challenges, particularly for biologists and clinicians who may lack engineering expertise. A major barrier is the reliance on specialized fabrication techniques, such as soft lithography and 3D printing, which are not typically available in standard cancer biology laboratories. While commercial availability could help bridge this gap, it requires standardized device components and operational workflows—both of which are currently lacking. Such standardization is crucial not only for reproducibility but also for enabling scalability and broader dissemination. To improve accessibility, future designs should focus on minimizing assembly complexity, providing clear, user-friendly manuals, and ensuring compatibility with conventional laboratory equipment such as microscopes, multiwell plates, and high-throughput screening platforms. By overcoming these barriers, lung/cancer-on-a-chip technologies can become more user-friendly, promoting wider adoption in cancer research and biomedical applications.

Finally, achieving complex lung cancer models for preclinical drug discovery and individualized therapy requires seamless collaboration among scientists, clinicians, and bioengineers.^143^ Current challenges and solutions in this multidiscipline cooperation include the following: (1) Divergent priorities: Scientists focus on understanding disease mechanisms and preclinical data, while clinicians prioritize immediate, evidence-based patient care.^144^ (2) Communication gaps: Discipline-specific knowledge hinders mutual understanding. (3) Logistical barriers: Limited access to clinical samples, funding competition, and physical separation of labs and clinics hinder integration. Shared biorepositories and data-sharing platforms, along with clinical research associates, may streamline collaboration. To overcome these obstacles, scientists are encouraged to become well-versed in clinical workflows and gain an understanding of clinical practice to accurately identify existing deficits that research can address. Similarly, clinicians must be educated in basic science to facilitate meaningful collaboration. Once a mutual understanding is established, the next step involves regular cross-disciplinary meetings to identify relevant research questions, exchange ideas, and monitor project progress.^144^ Addressing these barriers with efficient communication, shared infrastructure, and aligned priorities will accelerate translational research and its impact on clinical outcomes.

## Data Availability

Data sharing is not applicable to this article as no new data were created or analyzed in this study.

## References

[1] H. Sung, J. Ferlay, R. L. Siegel *et al.*, “Global cancer statistics 2020: GLOBOCAN estimates of incidence and mortality worldwide for 36 cancers in 185 countries,” CA Cancer J. Clin. 71(3), 209–249 (2021). 10.3322/caac.21660 33538338

[2] F. Bray, M. Laversanne, H. Sung *et al.*, “Global cancer statistics 2022: GLOBOCAN estimates of incidence and mortality worldwide for 36 cancers in 185 countries,” CA Cancer J. Clin. 74(3), 229–263 (2024). 10.3322/caac.21834 38572751

[3] R. S. Herbst, D. Morgensztern, and C. Boshoff, “The biology and management of non-small cell lung cancer,” Nature 553(7689), 446–454 (2018). 10.1038/nature25183 29364287

[4] A. Onaciu, R. Munteanu, V. C. Munteanu *et al.*, “Spontaneous and induced animal models for cancer research,” Diagnostics 10(9), 660 (2020). 10.3390/diagnostics10090660 32878340 PMC7555044

[5] G. Vlachogiannis, S. Hedayat, A. Vatsiou *et al.*, “Patient-derived organoids model treatment response of metastatic gastrointestinal cancers,” Science 359(6378), 920–926 (2018). 10.1126/science.aao2774 29472484 PMC6112415

[6] S. E. Park, A. Georgescu, and D. Huh, “Organoids-on-a-chip,” Science 364(6444), 960–965 (2019). 10.1126/science.aaw7894 31171693 PMC7764943

[7] H. J. Kim, S. Park, S. Jeong, J. Kim, and Y. J. Cho, “Lung organoid on a chip: A new ensemble model for preclinical studies,” Int. J. Stem Cells 17(1), 30–37 (2024). 10.15283/ijsc23090 37816583 PMC10899883

[8] B. Chen, C. Du, M. Wang, J. Guo, and X. Liu, “Organoids as preclinical models of human disease: Progress and applications,” Med. Rev. 4, 129 (2024). 10.1515/mr-2023-0047 PMC11046574 38680680

[9] A. Shah, J. Apple, A. J. Belli *et al.*, “Real-world study of disease-free survival and patient characteristics associated with disease-free survival in early-stage non-small cell lung cancer: A retrospective observational study,” Cancer Treat. Res. Commun. 36, 100742 (2023). 10.1016/j.ctarc.2023.100742 37478531

[10] R. L. Siegel, K. D. Miller, N. S. Wagle, and A. Jemal, “Cancer statistics, 2023,” CA Cancer J. Clin. 73(1), 17–48 (2023). 10.3322/caac.21763 36633525

[11] R. Rajaram, Q. Huang, R. Z. Li *et al.*, “Recurrence-free survival in patients with surgically resected non-small cell lung cancer: A systematic literature review and meta-analysis,” Chest 165, 1260–1270 (2024). 10.1016/j.chest.2023.11.042 38065405

[12] H. Sugimura, F. C. Nichols, P. Yang *et al.*, “Survival after recurrent nonsmall-cell lung cancer after complete pulmonary resection,” Ann. Thorac. Surg. 83(2), 409–418 (2007). 10.1016/j.athoracsur.2006.08.046 17257962

[13] A. L. Potter, P. Senthil, A. Keshwani *et al.*, “Long-term survival after lung cancer resection in the national lung screening trial,” Ann. Thorac. Surg. 117, 734–742 (2024). 10.1016/j.athoracsur.2023.12.011 38216080

[14] A. Casal-Mouriño, A. Ruano-Ravina, M. Lorenzo-González *et al.*, “Epidemiology of stage III lung cancer: Frequency, diagnostic characteristics, and survival,” Transl. Lung Cancer Res. 10(1), 506–518 (2021). 10.21037/tlcr.2020.03.40 33569332 PMC7867742

[15] S. Verfaillie, M. Lambrecht, P. Berkovic *et al.*, “Treatment of unresectable stage III NSCLC: Real world cohort study and literature review,” Cancer Treat. Res. Commun. 36, 100727 (2023). 10.1016/j.ctarc.2023.100727 37307680

[16] N. Martini, M. S. Bains, M. E. Burt *et al.*, “Incidence of local recurrence and second primary tumors in resected stage I lung cancer,” J. Thorac. Cardiovasc. Surg. 109, 120–129 (1995). 10.1016/S0022-5223(95)70427-2 7815787

[17] The Writing Committee for the International Adjuvant Lung Cancer Trial (IALT), R. Arriagada, I. De Radiomedic-Ina, C. Santiago *et al.* “Cisplatin-based adjuvant chemotherapy in patients with completely resected non-small-cell lung cancer,” N. Engl. J. Med. 350(4), 351–360 (2004). 10.1056/NEJMoa031644 14736927

[18] J. P. Pignon, H. Tribodet, G. V. Scagliotti *et al.*, “Lung adjuvant cisplatin evaluation: A pooled analysis by the LACE collaborative group,” J. Clin. Oncol. 26(21), 3552–3559 (2008). 10.1200/JCO.2007.13.9030 18506026

[19] K. L. Kehl, D. Zahrieh, P. Yang *et al.*, “Rates of guideline-concordant surgery and adjuvant chemotherapy among patients with early-stage lung cancer in the US ALCHEMIST study (Alliance A151216),” JAMA Oncol. 8(5), 717–728 (2022). 10.1001/jamaoncol.2022.0039 35297944 PMC8931674

[20] A. Cooper, J. E. Chaft, and M. J. Bott, “Induction therapy for non-small cell lung cancer,” J. Thorac. Cardiovasc. Surg. 168(2), 411–416 (2024). 10.1016/j.jtcvs.2024.01.048 38354767

[21] P. M. Forde, J. Spicer, S. Lu *et al.*, “Neoadjuvant nivolumab plus chemotherapy in resectable lung cancer,” N. Engl. J. Med. 386(21), 1973–1985 (2022). 10.1056/NEJMoa2202170 35403841 PMC9844511

[22] H. Wakelee, M. Liberman, T. Kato *et al.*, “Perioperative pembrolizumab for early-stage non-small-cell lung cancer,” N. Engl. J. Med. 389(6), 491–503 (2023). 10.1056/NEJMoa2302983 37272513 PMC11074923

[23] T. Cascone, M. M. Awad, J. D. Spicer *et al.*, “LBA1 CheckMate 77T: Phase III study comparing neoadjuvant nivolumab (NIVO) plus chemotherapy (chemo) vs neoadjuvant placebo plus chemo followed by surgery and adjuvant NIVO or placebo for previously untreated, resectable stage II–IIIb NSCLC,” Ann. Oncol. 34, S1295 (2023). 10.1016/j.annonc.2023.10.050

[24] E. Felip, N. Altorki, C. Zhou *et al.*, “Adjuvant atezolizumab after adjuvant chemotherapy in resected stage IB–IIIA non-small-cell lung cancer (IMpower010): A randomised, multicentre, open-label, phase 3 trial,” Lancet 398(10308), 1344–1357 (2021). 10.1016/S0140-6736(21)02098-5 34555333

[25] M. O'Brien, L. Paz-Ares, S. Marreaud *et al.*, “Pembrolizumab versus placebo as adjuvant therapy for completely resected stage IB–IIIA non-small-cell lung cancer (PEARLS/KEYNOTE-091): An interim analysis of a randomised, triple-blind, phase 3 trial,” Lancet Oncol. 23(10), 1274–1286 (2022). 10.1016/S1470-2045(22)00518-6 36108662

[26] Y. L. Wu, M. Tsuboi, J. He *et al.*, “Osimertinib in resected EGFR-mutated non-small-cell lung cancer,” N. Engl. J. Med. 383(18), 1711–1723 (2020). 10.1056/NEJMoa2027071 32955177

[27] M. Tsuboi, R. S. Herbst, T. John *et al.*, “Overall survival with osimertinib in resected EGFR-mutated NSCLC,” N. Engl. J. Med. 389(2), 137–147 (2023). 10.1056/NEJMoa2304594 37272535

[28] M. Tsuboi, W. Weder, C. Escriu *et al.*, “Neoadjuvant osimertinib with/without chemotherapy versus chemotherapy alone for EGFR-mutated resectable non-small-cell lung cancer: NeoADAURA,” Future Oncol. 17(31), 4045–4055 (2021). 10.2217/fon-2021-0549 34278827 PMC8530153

[29] A. Leonetti, R. Minari, L. Boni *et al.*, “Phase II, open-label, single-arm, multicenter study to assess the activity and safety of alectinib as neoadjuvant treatment in surgically resectable stage III ALK-positive NSCLC: ALNEO trial,” Clin. Lung Cancer 22(5), 473–477 (2021). 10.1016/j.cllc.2021.02.014 33762169

[30] M. Araghi, R. Mannani, A. Heidarnejad maleki *et al.*, “Recent advances in non-small cell lung cancer targeted therapy; an update review,” Cancer Cell Int. 23(1), 162 (2023). 10.1186/s12935-023-02990-y 37568193 PMC10416536

[31] D. Romaniello, I. Marrocco, N. B. Nataraj *et al.*, “Targeting HER3, a catalytically defective receptor tyrosine kinase, prevents resistance of lung cancer to a third-generation EGFR kinase inhibitor,” Cancers 12(9), 2394 (2020). 10.3390/cancers12092394 32847130 PMC7563838

[32] Y. Li, B. Yan, and S. He, “Advances and challenges in the treatment of lung cancer,” Biomed. Pharmacother. 169, 115891 (2023). 10.1016/j.biopha.2023.115891 37979378

[33] J. Lategahn, M. Keul, and D. Rauh, “Lessons to be learned: The molecular basis of kinase-targeted therapies and drug resistance in non-small cell lung cancer,” Angew. Chem., Int. Ed. 57(9), 2307–2313 (2018). 10.1002/anie.201710398 29178586

[34] J. L. Schneider, J. J. Lin, and A. T. Shaw, “ALK-positive lung cancer: A moving target,” Nat. Cancer 4(3), 330–343 (2023). 10.1038/s43018-023-00515-0 36797503 PMC10754274

[35] G. M. Whitesides, “The origins and the future of microfluidics,” Nature 442(7101), 368–373 (2006). 10.1038/nature05058 16871203

[36] M. Xie, Z. Zhan, Y. Li *et al.*, “Functional microfluidics: Theory, microfabrication, and applications,” Int. J. Extreme Manuf. 6(3), 032005 (2024). 10.1088/2631-7990/ad2c5f

[37] K. Wang, K. Man, J. Liu *et al.*, “Microphysiological systems: Design, fabrication, and applications,” ACS Biomater. Sci. Eng. 6(6), 3231–3257 (2020). 10.1021/acsbiomaterials.9b01667 33204830 PMC7668566

[38] S. Nadine, A. Chung, S. E. Diltemiz *et al.*, “Advances in microfabrication technologies in tissue engineering and regenerative medicine,” Artif. Organs 46(7), E211–E243 (2022). 10.1111/aor.14232 35349178

[39] G. Velve-Casquillas, M. Le Berre, M. Piel, and P. T. Tran, “Microfluidic tools for cell biological research,” Nano Today 5(1), 28–47 (2010). 10.1016/j.nantod.2009.12.001 21152269 PMC2998071

[40] I. Miranda, A. Souza, P. Sousa *et al.*, “Properties and applications of PDMS for biomedical engineering: A review,” J. Funct. Biomater. 13(1), 2 (2021). 10.3390/jfb13010002 35076525 PMC8788510

[41] V. Velasco, S. A. Shariati, and R. Esfandyarpour, “Microtechnology-based methods for organoid models,” Microsyst. Nanoeng. 6(1), 76 (2020). 10.1038/s41378-020-00185-3 34567686 PMC8433138

[42] Q. Tang, X. Yang, C. Xuan, K. Wu, C. Lai, and X. Shi, “Generation of microfluidic gradients and their effects on cells behaviours,” Biodes. Manuf. 3(4), 427–431 (2020). 10.1007/s42242-020-00093-5

[43] A. J. Ozga, M. T. Chow, and A. D. Luster, “Chemokines and the immune response to cancer,” Immunity 54(5), 859–874 (2021). 10.1016/j.immuni.2021.01.012 33838745 PMC8434759

[44] P. E. Oomen, M. D. Skolimowski, and E. Verpoorte, “Implementing oxygen control in chip-based cell and tissue culture systems,” Lab Chip 16(18), 3394–3414 (2016). 10.1039/C6LC00772D 27492338

[45] C. Bouquerel, A. Dubrova, I. Hofer *et al.*, “Bridging the gap between tumor-on-chip and clinics: A systematic review of 15 years of studies,” Lab Chip 23, 3906 (2023). 10.1039/D3LC00531C 37592893

[46] M. D. Brennan, M. L. Rexius-Hall, L. J. Elgass, and D. T. Eddington, “Oxygen control with microfluidics,” Lab Chip 14(22), 4305–4318 (2014). 10.1039/C4LC00853G 25251498

[47] B. A. Hassell, G. Goyal, E. Lee *et al.*, “Human organ chip models recapitulate orthotopic lung cancer growth, therapeutic responses, and tumor dormancy in vitro,” Cell Rep. 21, 508–516 (2017). 10.1016/j.celrep.2017.09.043 29020635

[48] A. Sontheimer-Phelps, B. A. Hassell, D. E. Ingber, and P. H. John, “Modelling cancer in microfluidic human organs-on-chips,” Nat. Rev. Cancer 19, 65 (2019). 10.1038/s41568-018-0104-6 30647431

[49] D. Huh, H. Fujioka, Y. C. Tung *et al.* “Acoustically detectable cellular-level lung injury induced by fluid mechanical stresses in microfluidic airway systems,” Proc. Natl. Acad. Sci. 104(48), 18886–18891 (2007). 10.1073/pnas.0610868104 18006663 PMC2141877

[50] O. Goksel, M. I. Sipahi, S. Yanasik *et al.*, “Comprehensive analysis of resilience of human airway epithelial barrier against short-term PM2.5 inorganic dust exposure using in vitro microfluidic chip and ex vivo human airway models,” Allergy 79, 2953 (2024). 10.1111/all.16179 38868934

[51] D. Huh, B. D. Matthews, A. Mammoto, M. Montoya-Zavala, H. Yuan Hsin, and D. E. Ingber, “Reconstituting organ-level lung functions on a chip,” Science 328(5986), 1662–1668 (2010). 10.1126/science.1188302 20576885 PMC8335790

[52] S. Lee, J. Lim, J. Yu, J. Ahn, Y. Lee, and N. L. Jeon, “Engineering tumor vasculature on an injection-molded plastic array 3D culture (IMPACT) platform,” Lab Chip 19(12), 2071–2080 (2019). 10.1039/C9LC00148D 31049508

[53] S. A. Langhans, “Three-dimensional in vitro cell culture models in drug discovery and drug repositioning,” Front. Pharmacol. 9, 6 (2018). 10.3389/fphar.2018.00006 29410625 PMC5787088

[54] S. R. Caliari and J. A. Burdick, “A practical guide to hydrogels for cell culture,” Nat. Methods 13(5), 405–414 (2016). 10.1038/nmeth.3839 27123816 PMC5800304

[55] Q. Luan, I. Pulido, A. Isagirre *et al.*, “Deciphering fibroblast-induced drug resistance in non-small cell lung carcinoma through patient-derived organoids in agarose microwells,” Lab Chip 24, 2025 (2024). 10.1039/D3LC01044A 38410967 PMC11209828

[56] N. Azizipour, R. Avazpour, M. H. Weber, M. Sawan, A. Ajji, and D. H. Rosenzweig, “Uniform tumor spheroids on surface-optimized microfluidic biochips for reproducible drug screening and personalized medicine,” Micromachines 13(4), 587 (2022). 10.3390/mi13040587 35457892 PMC9028696

[57] D. J. Jung, T. H. Shin, M. Kim, C. O. Sung, S. J. Jang, and G. S. Jeong, “A one-stop microfluidic-based lung cancer organoid culture platform for testing drug sensitivity,” Lab Chip 19(17), 2854–2865 (2019). 10.1039/C9LC00496C 31367720

[58] S. W. Lee, S. Hong, B. Jung *et al.*, “In vitro lung cancer multicellular tumor spheroid formation using a microfluidic device,” Biotechnol. Bioeng. 116(11), 3041–3052 (2019). 10.1002/bit.27114 31294818

[59] Z. Xu, Y. Gao, Y. Hao *et al.*, “Application of a microfluidic chip-based 3D co-culture to test drug sensitivity for individualized treatment of lung cancer,” Biomaterials 34(16), 4109–4117 (2013). 10.1016/j.biomaterials.2013.02.045 23473962

[60] C. Zhang, K. Tian, Z. Meng *et al.*, “A versatile dilution-treatment-detection microfluidic chip platform for rapid In vitro lung cancer drug combination sensitivity evaluation,” Talanta 277, 126298 (2024). 10.1016/j.talanta.2024.126298 38823330

[61] M. Gao, W. Ding, Y. Wang *et al.*, “Quantitatively evaluating interactions between patient-derived organoids and autologous immune cells by microfluidic chip,” Anal. Chem. 96(32), 13061–13069 (2024). 10.1021/acs.analchem.4c01389 39093612

[62] X. Wu, B. Li, Y. Wang *et al.*, “Microfluidic chip-based automatic system for sequencing patient-derived organoids at the single-cell level,” Anal. Chem. 96, 17027–17048 (2024). 10.1021/acs.analchem.4c05111 39399894

[63] E. Purev, K. Bahmed, and B. Kosmider, “Alveolar organoids in lung disease modeling,” Biomolecules 14(1), 115 (2024). 10.3390/biom14010115 38254715 PMC10813493

[64] X. Sun, A. K. Perl, R. Li *et al.*, “A census of the lung: CellCards from LungMAP,” Dev. Cell 57(1), 112–145.e2 (2022). 10.1016/j.devcel.2021.11.007 34936882 PMC9202574

[65] A. Gillich, F. Zhang, C. G. Farmer *et al.*, “Capillary cell-type specialization in the alveolus,” Nature 586(7831), 785–789 (2020). 10.1038/s41586-020-2822-7 33057196 PMC7721049

[66] J. Wang, X. Li, and H. Chen, “Organoid models in lung regeneration and cancer,” Cancer Lett. 475, 129–135 (2020). 10.1016/j.canlet.2020.01.030 32032677

[67] W. K. C. Cheung and D. X. Nguyen, “Lineage factors and differentiation states in lung cancer progression,” Oncogene 34(47), 5771–5780 (2015). 10.1038/onc.2015.85 25823023 PMC7597437

[68] C. Blanpain, “Tracing the cellular origin of cancer,” Nat. Cell Biol. 15(2), 126–134 (2013). 10.1038/ncb2657 23334500

[69] K. D. Sutherland, N. Proost, I. Brouns, D. Adriaensen, J. Y. Song, and A. Berns, “Cell of origin of small cell lung cancer: Inactivation of Trp53 and Rb1 in distinct cell types of adult mouse lung,” Cancer Cell 19(6), 754–764 (2011). 10.1016/j.ccr.2011.04.019 21665149

[70] G. Ferone, J. Y. Song, K. D. Sutherland *et al.*, “SOX2 is the determining oncogenic switch in promoting lung squamous cell carcinoma from different cells of origin,” Cancer Cell 30(4), 519–532 (2016). 10.1016/j.ccell.2016.09.001 27728803 PMC5065004

[71] T. J. Desai, D. G. Brownfield, and M. A. Krasnow, “Alveolar progenitor and stem cells in lung development, renewal and cancer,” Nature 507(7491), 190–194 (2014). 10.1038/nature12930 24499815 PMC4013278

[72] Z. Li, X. Zhuang, C. H. Pan *et al.*, “Alveolar differentiation drives resistance to KRAS inhibition in lung adenocarcinoma,” Cancer Discovery 14(2), 308–325 (2024). 10.1158/2159-8290.CD-23-0289 37931288 PMC10922405

[73] X. Han, T. Na, T. Wu, and B. Z. Yuan, “Human lung epithelial BEAS-2B cells exhibit characteristics of mesenchymal stem cells,” PLoS One 15(1), e0227174 (2020). 10.1371/journal.pone.0227174 31900469 PMC6941928

[74] J. L. Kerschner, A. Paranjapye, and A. Harris, “Cellular heterogeneity in the 16HBE14o^−^ airway epithelial line impacts biological readouts,” Physiol. Rep. 11(11), e15700 (2023). 10.14814/phy2.15700 37269165 PMC10238858

[75] K. L. Sellgren, E. J. Butala, B. P. Gilmour, S. H. Randell, and S. Grego, “A biomimetic multicellular model of the airways using primary human cells,” Lab Chip 14(17), 3349–3358 (2014). 10.1039/C4LC00552J 25000964

[76] J. D. Stucki, N. Hobi, A. Galimov *et al.*, “Medium throughput breathing human primary cell alveolus-on-chip model,” Sci. Rep. 8(1), 14359 (2018). 10.1038/s41598-018-32523-x 30254327 PMC6156575

[77] P. Zamprogno, S. Wüthrich, S. Achenbach *et al.*, “Second-generation lung-on-a-chip with an array of stretchable alveoli made with a biological membrane,” Commun. Biol. 4(1), 168 (2021). 10.1038/s42003-021-01695-0 33547387 PMC7864995

[78] M. Humayun, C. W. Chow, and E. W. K. Young, “Microfluidic lung airway-on-a-chip with arrayable suspended gels for studying epithelial and smooth muscle cell interactions,” Lab Chip 18(9), 1298–1309 (2018). 10.1039/C7LC01357D 29651473

[79] A. Jain, R. Barrile, A. D. Van Der Meer *et al.*, “A primary human lung alveolus-on-a-chip model of intravascular thrombosis for assessment of therapeutics,” Clin. Pharmacol. Ther. 103(2), 332–340 (2018). 10.1002/cpt.742 28516446 PMC5693794

[80] K. H. Benam, R. Villenave, C. Lucchesi *et al.*, “Small airway-on-a-chip enables analysis of human lung inflammation and drug responses in vitro,” Nat. Methods 13(2), 151 (2016). 10.1038/nmeth.3697 26689262

[81] E. K. Min, C. M. Lee, S. R. Kim *et al.*, “Advanced lung-on-a-chip technology: Mimicking the complex human lung microenvironment,” Int. J. Biol. Sci. 21(1), 17–39 (2025). 10.7150/ijbs.105702 39744426 PMC11667821

[82] S. L. Faley, N. A. Boghdeh, D. K. Schaffer *et al.*, “Gravity-perfused airway-on-a-chip optimized for quantitative BSL-3 studies of SARS-CoV-2 infection: Barrier permeability, cytokine production, immunohistochemistry, and viral load assays,” Lab Chip 24(6), 1794–1807 (2024). 10.1039/D3LC00894K 38362777 PMC10929697

[83] X. Y. Tang, S. Wu, D. Wang *et al.*, “Human organoids in basic research and clinical applications,” Signal Transduction Targeted Ther. 7(1), 168 (2022). 10.1038/s41392-022-01024-9 PMC9127490 35610212

[84] K. T. Leeman, C. M. Fillmore, and C. F. Kim, “Lung stem and progenitor cells in tissue homeostasis and disease,” Curr. Top. Dev. Biol. 107, 207–233 (2014). 10.1016/B978-0-12-416022-4.00008-1 24439808 PMC4038302

[85] K. Hoffmann, B. Obermayer, K. Hönzke *et al.*, “Human alveolar progenitors generate dual lineage bronchioalveolar organoids,” Commun. Biol. 5(1), 875 (2022). 10.1038/s42003-022-03828-5 36008580 PMC9409623

[86] M. C. Basil, J. Katzen, A. E. Engler *et al.*, “The cellular and physiological basis for lung repair and regeneration: Past, present, and future,” Cell Stem Cell 26(4), 482–502 (2020). 10.1016/j.stem.2020.03.009 32243808 PMC7128675

[87] T. Thangam, K. Parthasarathy, K. Supraja *et al.*, “Lung organoids: Systematic review of recent advancements and its future perspectives,” Tissue Eng. Regener. Med. 21(5), 653–671 (2024). 10.1007/s13770-024-00628-2 PMC11187038 38466362

[88] S. Gotoh, I. Ito, T. Nagasaki *et al.*, “Generation of alveolar epithelial spheroids via isolated progenitor cells from human pluripotent stem cells,” Stem Cell Rep. 3(3), 394–403 (2014). 10.1016/j.stemcr.2014.07.005 PMC4266003 25241738

[89] A. Jacob, M. Morley, F. Hawkins *et al.*, “Differentiation of human pluripotent stem cells into functional lung alveolar epithelial cells,” Cell Stem Cell 21(4), 472–488.e10 (2017). 10.1016/j.stem.2017.08.014 28965766 PMC5755620

[90] S. X. L. Huang, M. N. Islam, J. O'Neill *et al.*, “Efficient generation of lung and airway epithelial cells from human pluripotent stem cells,” Nat. Biotechnol. 32(1), 84–91 (2014). 10.1038/nbt.2754 24291815 PMC4101921

[91] K. B. McCauley, F. Hawkins, M. Serra, D. C. Thomas, A. Jacob, and D. N. Kotton, “Efficient derivation of functional human airway epithelium from pluripotent stem cells via temporal regulation of Wnt signaling,” Cell Stem Cell 20(6), 844–857.e6 (2017). 10.1016/j.stem.2017.03.001 28366587 PMC5457392

[92] M. Serra, K. D. Alysandratos, F. Hawkins *et al.*, “Pluripotent stem cell differentiation reveals distinct developmental pathways regulating lung-versus thyroid-lineage specification,” Development 144(21), 3879–3893 (2017). 10.1242/dev.150193 28947536 PMC5702071

[93] F. Hawkins, P. Kramer, A. Jacob *et al.*, “Prospective isolation of NKX2-1-expressing human lung progenitors derived from pluripotent stem cells,” J. Clin. Invest. 127(6), 2277–2294 (2017). 10.1172/JCI89950 28463226 PMC5451263

[94] Y. Korogi, S. Gotoh, S. Ikeo *et al.*, “In vitro disease modeling of Hermansky-Pudlak syndrome type 2 using human induced pluripotent stem cell-derived alveolar organoids,” Stem Cell Rep. 12(3), 431–440 (2019). 10.1016/j.stemcr.2019.01.014 PMC6409438 30773483

[95] N. V. Dorrello, B. A. Guenthart, J. D. O'neill *et al.*, “Functional vascularized lung grafts for lung bioengineering,” Sci. Adv. 3(8), e1700521 (2017). 10.1126/sciadv.1700521 28875163 PMC5576878

[96] N. K. Altorki, G. J. Markowitz, D. Gao *et al.*, “The lung microenvironment: An important regulator of tumour growth and metastasis,” Nat. Rev. Cancer 19(1), 9–31 (2019). 10.1038/s41568-018-0081-9 30532012 PMC6749995

[97] A. M. G. Houghton, “Mechanistic links between COPD and lung cancer,” Nat. Rev. Cancer 13(4), 233–245 (2013). 10.1038/nrc3477 23467302

[98] M. Ito, G. Ishii, K. Nagai, R. Maeda, Y. Nakano, and A. Ochiai, “Prognostic impact of cancer-associated stromal cells in patients with stage I lung adenocarcinoma,” Chest 142(1), 151–158 (2012). 10.1378/chest.11-2458 22302300

[99] K. Reynders and D. De Ruysscher, “Tumor infiltrating lymphocytes in lung cancer: A new prognostic parameter,” J. Thorac. Dis. 8(8), E833–E835 (2016). 10.21037/jtd.2016.07.75 27618931 PMC4999748

[100] K. Shimizu, M. Nakata, Y. Hirami, T. Yukawa, A. Maeda, and K. Tanemoto, “Tumor-infiltrating Foxp3+ regulatory T cells are correlated with cyclooxygenase-2 expression and are associated with recurrence in resected non-small cell lung cancer,” J. Thorac. Oncol. 5(5), 585–590 (2010). 10.1097/JTO.0b013e3181d60fd7 20234320

[101] D. A. Close, A. X. Wang, S. J. Kochanek, T. Shun, J. L. Eiseman, and P. A. Johnston, “Implementation of the NCI-60 human tumor cell line panel to screen 2260 cancer drug combinations to generate >3 million data points used to populate a large matrix of anti-neoplastic agent combinations (ALMANAC) database,” SLAS Discovery 24(3), 242–263 (2019). 10.1177/2472555218812429 30500310

[102] K. Furugaki, M. Mochizuki, M. Kohno, S. Shu, N. Harada, and Y. Yoshimura, “Expression of C-terminal ALK, RET, or ROS1 in lung cancer cells with or without fusion,” BMC Cancer 19(1), 301 (2019). 10.1186/s12885-019-5527-2 30943926 PMC6446279

[103] S. Gunti, A. T. K. Hoke, K. P. Vu, and N. R. London, “Organoid and spheroid tumor models: Techniques and applications,” Cancers 13(4), 874 (2021). 10.3390/cancers13040874 33669619 PMC7922036

[104] C. M. Della Corte, G. Barra, V. Ciaramella *et al.*, “Antitumor activity of dual blockade of PD-L1 and MEK in NSCLC patients derived three-dimensional spheroid cultures,” J. Exp. Clin. Cancer Res. 38(1), 253 (2019). 10.1186/s13046-019-1257-1 31196138 PMC6567578

[105] W. Liao, J. Wang, J. Xu *et al.*, “High-throughput three-dimensional spheroid tumor model using a novel stamp-like tool,” J. Tissue Eng. 10, 2041731419889184 (2019). 10.1177/2041731419889184 31827757 PMC6886283

[106] A. B. Holton, F. L. Sinatra, J. Kreahling, A. J. Conway, D. A. Landis, and S. Altiok, “Microfluidic biopsy trapping device for the real-time monitoring of tumor microenvironment,” PLoS One 12(1), e0169797 (2017). 10.1371/journal.pone.0169797 28085924 PMC5235371

[107] L. Cords, S. Engler, M. Haberecker *et al.*, “Cancer-associated fibroblast phenotypes are associated with patient outcome in non-small cell lung cancer,” Cancer Cell 42(3), 396–412.e5 (2024). 10.1016/j.ccell.2023.12.021 38242124 PMC10929690

[108] J. Kim, H. Park, H. Kim, Y. T. Kim, H. J. Oh, and S. Chung, “Microfluidic one-directional interstitial flow generation from cancer to cancer associated fibroblast,” Acta Biomater. 144, 258–265 (2022). 10.1016/j.actbio.2022.03.044 35364320

[109] T. Yasuda, M. Koiwa, A. Yonemura, T. Akiyama, H. Baba, and T. Ishimoto, “Protocol to establish cancer-associated fibroblasts from surgically resected tissues and generate senescent fibroblasts,” STAR Protoc. 2(2), 100553 (2021). 10.1016/j.xpro.2021.100553 34136831 PMC8176369

[110] H. T. Tzeng and Y. J. Huang, “Tumor vasculature as an emerging pharmacological target to promote anti-tumor immunity,” Int. J. Mol. Sci. 24(5), 4422 (2023). 10.3390/ijms24054422 36901858 PMC10002465

[111] M. B. Schaaf, A. D. Garg, and P. Agostinis, “Defining the role of the tumor vasculature in antitumor immunity and immunotherapy article,” Cell Death Dis. 9(2), 115 (2018). 10.1038/s41419-017-0061-0 29371595 PMC5833710

[112] Z. Xu, E. Li, Z. Guo *et al.*, “Design and construction of a multi-organ microfluidic chip mimicking the in vivo microenvironment of lung cancer metastasis,” ACS Appl. Mater. Interfaces 8(39), 25840–25847 (2016). 10.1021/acsami.6b08746 27606718

[113] S. Park, T. H. Kim, S. H. Kim, S. You, and Y. Jung, “Three-dimensional vascularized lung cancer-on-a-chip with lung extracellular matrix hydrogels for in vitro screening,” Cancers 13(16), 3930 (2021). 10.3390/cancers13163930 34439103 PMC8393390

[114] C. Quintard, E. Tubbs, G. Jonsson *et al.*, “A microfluidic platform integrating functional vascularized organoids-on-chip,” Nat. Commun. 15(1), 1452 (2024). 10.1038/s41467-024-45710-4 38365780 PMC10873332

[115] T. Yu, Q. Yang, B. Peng, Z. Gu, and D. Zhu, “Vascularized organoid-on-a-chip: Design, imaging, and analysis,” Angiogenesis 27, 147–172 (2024). 10.1007/s10456-024-09905-z 38409567

[116] Z. Zhou, Y. Pang, J. Ji *et al.*, “Harnessing 3D in vitro systems to model immune responses to solid tumours: A step towards improving and creating personalized immunotherapies,” Nat. Rev. Immunol. 24, 18–32 (2024). 10.1038/s41577-023-00896-4 37402992

[117] L. Arora, M. Kalia, S. Dasgupta, N. Singh, A. K. Verma, and D. Pal, “Development of a multicellular 3D tumor model to study cellular heterogeneity and plasticity in NSCLC tumor microenvironment,” Front Oncol. 12, 881207 (2022). 10.3389/fonc.2022.881207 35837091 PMC9273950

[118] Y. Zhang, Q. Hu, Y. Pei *et al.*, “A patient-specific lung cancer assembloid model with heterogeneous tumor microenvironments,” Nat. Commun. 15(1), 3382 (2024). 10.1038/s41467-024-47737-z 38643164 PMC11032376

[119] P. Sardarabadi, K. Y. Lee, W. L. Sun, A. A. Kojabad, and C. H. Liu, “Investigating T cell immune dynamics and IL-6's duality in a microfluidic lung tumor model,” ACS Appl. Mater. Interfaces 17, 4354 (2025). 10.1021/acsami.4c09065 39471283 PMC11758792

[120] G. Hamilton, B. Rath, and S. Stickler, “Significance of circulating tumor cells in lung cancer: A narrative review,” Transl. Lung Cancer Res. 12(4), 877–894 (2023). 10.21037/tlcr-22-712 37197632 PMC10183408

[121] J. Zhou, A. Kulasinghe, A. Bogseth, K. O'byrne, C. Punyadeera, and I. Papautsky, “Isolation of circulating tumor cells in non-small-cell-lung-cancer patients using a multi-flow microfluidic channel,” Microsyst. Nanoeng. 5(1), 8 (2019). 10.1038/s41378-019-0045-6 31057935 PMC6387977

[122] Z. Qiao, X. Teng, A. Liu, and W. Yang, “Novel isolating approaches to circulating tumor cell enrichment based on microfluidics: A review,” Micromachines 15(6), 706 (2024). 10.3390/mi15060706 38930676 PMC11206030

[123] C. B. Meador and A. N. Hata, “Acquired resistance to targeted therapies in NSCLC: Updates and evolving insights,” Pharmacol. Ther. 210, 107522 (2020). 10.1016/j.pharmthera.2020.107522 32151666 PMC8675642

[124] L. Huang, Y. Xu, N. Wang *et al.*, “Next-generation preclinical functional testing models in cancer precision medicine: CTC-derived organoids,” Small Methods 8(1), 2301009 (2024). 10.1002/smtd.202301009 37882328

[125] M. Yu, A. Bardia, N. Aceto *et al.*, “Ex vivo culture of circulating breast tumor cells for individualized testing of drug susceptibility,” Science 345, 216 (2014). www.sciencemag.org 10.1126/science.1253533 25013076 PMC4358808

[126] Z. Zhang, H. Shiratsuchi, J. Lin *et al.*, “Expansion of CTCs from early stage lung cancer patients using a microfluidic co-culture model,” Oncotarget 5, 12383 (2014). www.impactjournals.com/oncotarget/10.18632/oncotarget.2592 25474037 PMC4323004

[127] B. L. Khoo, G. Grenci, Y. B. Lim, S. C. Lee, J. Han, and C. T. Lim, “Expansion of patient-derived circulating tumor cells from liquid biopsies using a CTC microfluidic culture device,” Nat. Protoc. 13(1), 34–58 (2018). 10.1038/nprot.2017.125 29215634

[128] C. Pan, X. Wang, C. Yang, K. Fu, F. Wang, and L. Fu, “The culture and application of circulating tumor cell-derived organoids,” Trends Cell Biol. 35, 364 (2025). 10.1016/j.tcb.2024.10.004 39523200

[129] N. Moore, D. Doty, M. Zielstorff *et al.*, “A multiplexed microfluidic system for evaluation of dynamics of immune-tumor interactions,” Lab Chip 18(13), 1844–1858 (2018). 10.1039/C8LC00256H 29796561

[130] D. Park, K. Son, Y. Hwang *et al.*, “High-throughput microfluidic 3D cytotoxicity assay for cancer immunotherapy (CACI-ImpacT platform),” Front Immunol. 10, 1133 (2019). 10.3389/fimmu.2019.01133 31191524 PMC6546835

[131] Y. Peng and E. Lee, “Microphysiological systems for cancer immunotherapy research and development,” Adv. Biol. 8(8), 2300077 (2024). 10.1002/adbi.202300077 PMC10770294 37409385

[132] H. Shigeto, E. Yamada, M. Kitamatsu *et al.*, “Analysis of single nucleotide-mutated single-cancer cells using the combined technologies of single-cell microarray chips and peptide nucleic acid-DNA probes,” Micromachines 11(7), 628 (2020). 10.3390/mi11070628 32605095 PMC7407912

[133] C. Shen, C. Zhan, Z. Tong *et al.*, “Detecting EGFR gene amplification using a fluorescence in situ hybridization platform based on digital microfluidics,” Talanta 269, 125444 (2024). 10.1016/j.talanta.2023.125444 38042143

[134] K. Zhang, J. Xi, H. Zha *et al.*, “A dual-functional microfluidic chip for guiding personalized lung cancer medicine: Combining EGFR mutation detection and organoid-based drug response test,” Lab Chip 24(6), 1762–1774 (2024). 10.1039/D3LC00974B 38352981

[135] D. Lee, Y. Kim, and C. Chung, “Scientific validation and clinical application of lung cancer organoids,” Cells 10(11), 3012 (2021). 10.3390/cells10113012 34831235 PMC8616085

[136] M. Kim, H. Mun, C. O. Sung *et al.*, “Patient-derived lung cancer organoids as in vitro cancer models for therapeutic screening,” Nat. Commun. 10(1), 3991 (2019). 10.1038/s41467-019-11867-6 31488816 PMC6728380

[137] J. Shi, Y. Pan, X. Liu, W. Cao, Y. Mu, and Q. Zhu, “Spatial omics sequencing based on microfluidic array chips,” Biosensors (Basel) 13(7), 712 (2023). 10.3390/bios13070712 37504111 PMC10377411

[138] J. Zhu, K. Pang, B. Hu *et al.*, “Custom microfluidic chip design enables cost-effective three-dimensional spatiotemporal transcriptomics with a wide field of view,” Nat. Genet. 56, 2259 (2024). 10.1038/s41588-024-01906-4 39256584 PMC11525186

[139] J. Li, Z. Wang, Q. Chu, K. Jiang, J. Li, and N. Tang, “The strength of mechanical forces determines the differentiation of alveolar epithelial cells,” Dev. Cell 44(3), 297–312.e5 (2018). 10.1016/j.devcel.2018.01.008 29408236

[140] A. Salem, M. C. Asselin, B. Reymen *et al.*, “Targeting hypoxia to improve non–small cell lung cancer outcome,” J. Natl. Cancer Inst. 110(1), 14–30 (2018). 10.1093/jnci/djx160 28922791

[141] M. G. Oser, D. MacPherson, T. G. Oliver, J. Sage, and K. S. Park, “Genetically-engineered mouse models of small cell lung cancer: The next generation,” Oncogene 43(7), 457–469 (2024). 10.1038/s41388-023-02929-7 38191672 PMC11180418

[142] A. F. M. Dost, A. L. Moye, M. Vedaie *et al.*, “Organoids model transcriptional hallmarks of oncogenic KRAS activation in lung epithelial progenitor cells,” Cell Stem Cell 27(4), 663–678.e8 (2020). 10.1016/j.stem.2020.07.022 32891189 PMC7541765

[143] D. M. G. Rubio, E. E. Schoenbaum, L. S. Lee *et al.*, “Defining translational research: Implications for training,” Acad. Med. 85(3), 470–475 (2010). 10.1097/ACM.0b013e3181ccd618 20182120 PMC2829707

[144] L. L. Restifo and G. R. Phelan, “The cultural divide: Exploring communication barriers between scientists and clinicians,” DMM Dis. Models Mech. 4(4), 423–426 (2011). 10.1242/dmm.008177 PMC3124044 21708897

